# Ecological divergence despite common mating sites: Genotypes and symbiotypes shed light on cryptic diversity in the black bean aphid species complex

**DOI:** 10.1038/s41437-024-00687-0

**Published:** 2024-05-14

**Authors:** Elena Gimmi, Jesper Wallisch, Christoph Vorburger

**Affiliations:** 1 Department of Aquatic Ecology, Eawag, Swiss Federal Institute of Aquatic Science and Technology, Dübendorf, Switzerland; 2 D-USYS, Department of Environmental Systems Science, ETH Zürich, Switzerland

**Keywords:** Ecological genetics, Population genetics, Evolutionary genetics

## Abstract

Different host plants represent ecologically dissimilar environments for phytophagous insects. The resulting divergent selection can promote the evolution of specialized host races, provided that gene flow is reduced between populations feeding on different plants. In black bean aphids belonging to the *Aphis fabae* complex, several morphologically cryptic taxa have been described based on their distinct host plant preferences. However, host choice and mate choice are largely decoupled in these insects: they are host-alternating and migrate between specific summer host plants and shared winter hosts, with mating occurring on the shared hosts. This provides a yearly opportunity for gene flow among aphids using different summer hosts, and raises the question if and to what extent the ecologically defined taxa are reproductively isolated. Here, we analyzed a geographically and temporally structured dataset of microsatellite genotypes from *A. fabae* that were mostly collected from their main winter host *Euonymus europaeus*, and additionally from another winter host and fourteen summer hosts. The data reveals multiple, strongly differentiated genetic clusters, which differ in their association with different summer and winter hosts. The clusters also differ in the frequency of infection with two heritable, facultative endosymbionts, separately hinting at reproductive isolation and divergent ecological selection. Furthermore, we found evidence for occasional hybridization among genetic clusters, with putative hybrids collected more frequently in spring than in autumn. This suggests that similar to host races in other phytophagous insects, both prezygotic and postzygotic barriers including selection against hybrids maintain genetic differentiation among *A. fabae* taxa, despite a common mating habitat.

## Introduction

Contrasting environments can impose differential selection on separate populations of a species, thereby causing ecologically based adaptive divergence. In this process of specialization, reduced gene flow and assortative mating may represent both drivers and effects of increasing population differentiation, and could eventually lay the ground for ecological speciation (e.g. Dobzhansky 1940; Rice 1987; Rundle and Nosil 2005; Schluter 2001). The evolution of separate, specialized species thereby represents the endpoint of a wide continuum of divergence, ranging from weak genetic differentiation to complete reproductive isolation between populations (Dobzhansky 1940; Nosil 2012; Schluter 2000). Among model organisms studied to investigate ecologically based population divergence and the potential of ecological speciation, phytophagous insects assume a prominent position (e.g. Berlocher and Feder 2002; Funk et al. 2002; Matsubayashi et al. 2010; Via 2001). Their host plants often represent habitat, food source, and mating site all in one, and the variable chemical and physical properties of different plant species may impose very specific selection pressures on the insects exploiting them. Examples of polyphagous insect species that appear structured into host-specialized lineages, often referred to as biotypes or host races, are abundant (Jaenike 1990), and novel examples are frequently discovered (e.g. Mlynarek and Heard 2018; Villacis-Perez et al. 2021). Specialization may be associated with variable amounts of genetic differentiation and reproductive compatibility between host races (Drès and Mallet 2002; Ehrlich and Murphy 1988; Harrison et al. 2022; Mitchell 1981), which makes them attractive models for exploring how the interplay of ecology and evolution shapes genetic structure within and among species (Berlocher and Feder 2002).

The evolution of host-specific insect lineages may be initiated by the physical separation of populations, for example following the acquisition of a new host species. This may result in reduced realized gene flow and facilitate adaptation to each host plant (Guldemond and Mackenzie 1994; Rice and Salt 1988). Specialized host lineages could also originate as a consequence of antagonistic pleiotropy or trade-offs regarding performance on different plants, favoring the linkage of performance and preference traits (Felsenstein 1982; Fry 2003; Futuyma and Peterson 1985; Jaenike 1978; Sandoval and Nosil 2005; Soudi et al. 2015). In either case, if different sets of alleles are responsible for adaptation to different plant species, offspring from parents specialized to different plants could experience reduced fitness due to their intermediate genotypes (Egan and Funk 2009; Thompson et al. 2019). This might promote the evolution of assortative mating (Howard 1993; Mackenzie and Guldemond 1994), thereby reinforcing reproductive isolation between populations.

A prime example of host plant-associated ecological specialization is the species complex formed by the pea aphid, *Acyrthosiphon pisum* (Hemiptera: Aphididae), a sap-sucking insect. *Ac. pisum* comprises multiple genetically distinct populations that differ in their preference for, and performance on, different legume genera (Fabaceae, Frantz et al. 2006; Peccoud et al. 2009; Simon et al. 2003; Via 1991). These host-associated populations typically also differ in the communities of facultative bacterial endosymbionts they harbor (Ferrari et al. 2012; Smith et al. 2015). In the pea aphid complex, host preference and host performance are heritable (Via 1991; Via 1999), the responsible loci seem to be linked (Hawthorne and Via 2001), and there is evidence for selection against both migrants and hybrids (Via et al. 2000). It appears that strong host fidelity, with individuals feeding and mating on the same plant species throughout their life cycle, provides a significant barrier to gene flow among pea aphid host races.

In contrast to *Ac. pisum*, a minority of aphid species are host alternating (dioecious): they undergo the sexual generation on a woody primary host plant species and most of the parthenogenetic generations on herbaceous secondary host plant species. A well-studied example of this dioecious lifestyle is the black bean aphid, *Aphis fabae*: females called fundatrices hatch in spring from overwintering eggs on the primary hosts (predominantly the European spindle tree, *Euonymus europaeus*, and the guelder-rose, *Viburnum opulus*). From there, their clonal offspring migrate to a large number of secondary hosts and reproduce parthenogenetically during summer (Blackman and Eastop 2000). In autumn, sexual males and females are produced and migrate back to the primary hosts, where they mate and lay overwintering eggs. Intriguingly, *A. fabae* also forms a complex of morphologically cryptic lineages, taxonomically treated as subspecies or species, which show a high degree of specialization to certain secondary host plant species, even though they meet and mate on common primary hosts (Blackman and Eastop 2000; Iglisch 1968; Müller 1982; Thieme 1987) (Table S1). The use of a shared mating habitat implies the potential for homogenizing gene flow among lineages, which may be counteracted by trade-offs in secondary host plant utilization (Mackenzie 1996), reduced hybrid fitness (Müller 1982; Tosh et al. 2004), or behavioral mechanisms (Raymond et al. 2001; Thieme and Dixon 1996). The understanding of the genetic structure of the *A. fabae* complex is still limited. While mitochondrial COI/II and CytB sequences reveal no clear genetic structure within the complex (Béji et al. 2015; Coeur d’acier et al. 2007; Coeur d’acier et al. 2014; Zhang et al. 2010), genetic differences have been found among multiple taxa using enzyme electrophoresis (Jörg and Lampel 1996). Furthermore, nuclear microsatellite markers revealed strong genetic differentiation between *A. fabae cirsiiacanthoides*, a taxon feeding on thistle (*Cirsium vulgare*) and *A. fabae fabae*, the nominal subspecies feeding on goosefoot (*Chenopodium album*) (Coeur d’acier et al. 2004; Vorburger et al. 2017). The fact that differentiation is revealed by unlinked, selectively neutral genetic markers indicates the presence of barriers to gene flow between certain members of the *A. fabae* complex. It suggests that, despite the use of a common mating site and the seeming lack of an environmental barrier to mating, host plant specialization of *A. fabae* is not (anymore) the sole result of heterogeneous selection on different summer hosts of one freely interbreeding population. However, the actual diversity of genetically diverging *A. fabae* lineages encountering each other on the common mating hosts remains unknown, as well as the extent of reproductive isolation among these.

The present work is based on an extensive, temporally and geographically structured dataset of *A. fabae* samples collected from their primary host plant *E. europaeus*. The samples were collected as part of a different study (Gimmi et al. 2023), which required identifying those individuals belonging to the nominal subspecies *A. f. fabae* by microsatellite genotyping. Here we analyzed this dataset more in depth with the goal of describing the genetic structure and diversity of *A. fabae* on *E. europaeus*. To put the original data into context, we complemented it with a collection of *A. fabae* individuals from the primary host plant *V. opulus* and from 14 different secondary host plants. We asked how many distinct genetic clusters we could identify among the collected black bean aphids, and whether individuals belonging to different genetic clusters are associated with distinct host plants. We also looked for evidence of hybridization among the distinct *A. fabae* lineages. Furthermore, since host race-specific endosymbiont communities are characteristic of various herbivorous insects and of the related pea aphid system in particular (Ferrari et al. 2012), we tested for the presence of the two maternally inherited, facultative bacterial endosymbionts *Hamiltonella defensa* and *Regiella insecticola* in all our aphid samples. These endosymbionts may provide different ecological functions including protection against pathogens and parasitoids (Feldhaar 2011; Guo et al. 2017; Oliver et al. 2010), but they also entail fitness costs (Polin et al. 2014; Vorburger and Gouskov 2011; Zytynska et al. 2021). Differing symbiont complements can thus be considered as an independent indication of population divergence and ecological specialization of their hosts (Ferrari et al. 2012; Hosokawa et al. 2007; Tsuchida et al. 2004).

## Methods

### The *Aphis fabae* complex

According to Blackman and Eastop (2017), the *Aphis fabae* complex comprises five taxa, *A.f. cirsiiacanthoides*, *A.f. fabae*, *A.f. mordwilkoi*, *A. evonymi*, and *A. solanella*, of which all but *A.f. mordwilkoi* use the European spindle tree, *E. europaeus*, as winter host. The guelder rose *V. opulus* and the mock orange *Philadelphus coronarius* are used as winter hosts by *A.f. mordwilkoi* and *A.f. cirsiiacanthoides. A. evonymi* does not host alternate but remains on *E. europaeus* throughout the year (Blackman and Eastop 2000; Lampel and Meier 2007), while the other taxa are heteroecious and use a wide range of cultivated and wild plants as summer hosts. Some of the plants are considered ‘diagnostic’ and are used to identify the different *A. fabae* taxa based on their acceptance of these as hosts (Müller 1982). An overview of the taxa and their host plants is presented in Table S1. Although slight morphological differences might exist between some *A. fabae* taxa (e.g. Müller and Steiner 1986), it is widely accepted that biological information on host plant preference should be considered to identify ‘black bean aphids’ beyond the general term *A. fabae* (Blackman and Eastop 2000; Heie 1986; Jörg and Lampel 1996; Lampel and Meier 2007; Müller 1982; Thieme 1987). Caution is also advised as some host plants are used by additional taxa that are not considered part of the *A. fabae* complex, but which are morphologically very similar (Table S1).

### Aphid samples

The dataset used in this study consists of two parts: the first part comprises black bean aphids collected exclusively from their primary host *E. europaeus*. These samples were originally collected for a different study (Gimmi et al. 2023) in March, April, and October of the years 2019 and 2020, and in April 2021. For each time point, approximately 80 aphids were sampled in each of three municipalities near Zurich, Switzerland, situated 10 to 30 km apart from each other: *Faellanden* (47° 22′ N 8° 38′ E), *Gossau* (47° 19′ N 8° 45′ E) and *Steinmaur* (47° 30′ N 8° 27′ E). The three sampling sites included cultivated fields of various crops interspersed with weeds serving as summer hosts of *A. fabae*, and they were structured by woody hedges containing *E. europaeus* and *V. opulus* (the third possible winter host, *P. coronarius*, is not native to our study area). Aphids were collected within a radius of 1–2 km of the indicated sampling point depending on host plant availability. Single females were collected from host plants located at least 3 m from each other to avoid collecting aphids originating from the same clonal colony. Only wingless or visibly reproducing winged aphids were collected (virginoparae in spring and summer, gynoparae or oviparae on the winter hosts in autumn), to avoid collecting migrants that would have stopped by but not settled on the plant. The exact sample sizes and sampling dates are provided in Table S2. The second part of our sample set was collected from various host plants at multiple sites close to our research institute, including *Faellanden*, *Gossau*, and *Steinmaur*, and the city of Zurich (47° 22’ 0.01” N, 8° 33’ 0” E). We collected individuals from the alternative winter host *V. opulus* in April 2020 and April 2021, and we collected individuals from the following summer hosts in summer 2021: *Achillea millefolium*, *Aegopodium podagraria*, *Anthriscus sylvestris, Arctium lappa, Beta vulgaris*, *Capsella bursa-pastoris*, *Chenopodium album, Cirsium vulgare, Cirsium arvense*, *Galium aparine*, *Galium mollugo*, *Matricaria chamomilla*, *Papaver rhoeas*, *Rumex obtusifolius* and *Tropaeolum majus*. Again, samples were collected based on host plant availability but always in at least two of the municipalities *Faellanden*, *Gossau*, *Steinmaur*, and *Zurich*, taking a single female aphid per host plant individual from host plants located at least 3 m from each other. Sample sizes ranged from 14 to 37 per summer host plant species (Table S2).

### DNA extraction and genotyping

Aphid DNA was extracted using a salting out protocol as in Sunnucks and Hales (1996). Each sample was genotyped at eight microsatellite loci (Af85, Af181, Af86, Af48, Af82, Afbeta, AfF, and Af50) using the primers of Coeur d’acier et al. (2004), which in previous studies showed no evidence of tight physical linkage (Sandrock et al. 2011) and proved to be reliable and successful in separating *A. f. fabae* and *A. f. cirsiiacanthoides* (Vorburger et al. 2017). Primer sequences and the PCR protocol are provided in Table S3. After PCR amplification, the microsatellite fragments were run on an ABI 3730 automated sequencer. GeneMarker 3.0.1 (SoftGenetics) was used to score the alleles. Samples were used for further analysis if the alleles of at least seven of the eight markers were successfully scored (1.4% missing data in the final dataset). In the original dataset, 16 aphid genotypes occurred twice and one genotype three times, and we kept only one sample of each genotype for further analysis. To help identify genetic clusters within our sample collection, we complemented our dataset with the genotypes of 30 samples that were clearly identified as either *A. f. fabae* or *A. f. cirsiiacanthoides* in Vorburger et al. (2017). The final dataset comprised 1619 aphid genotypes from *E. europaeus* and 480 from other host plants, i.e. 2099 genotypes in total (Table S2). Allele numbers per locus varied from seven to 58 (Table S6).

### Analysis of genetic structure

All analyses using R were performed in Rstudio 2022.02.3 (RStudio Team 2020) with R 4.2.3 (R Core Team 2019) and using *ggplot2* 3.3.5 (Wickham 2016) for plotting. To assess the genetic structure present in our data and to assign samples to genetic clusters, we considered the results of three different clustering methods: *snapclust* (Beugin et al. 2018) implemented in the R package *adegenet* 2.1.5 (Jombart 2008), STRUCTURE 2.3.4 (Falush et al. 2003; Pritchard et al. 2000), and DAPC (Jombart et al. 2010) implemented in *adegenet* as well. *Snapclust* applies a combination of geometric and model-based steps and the Expectation-Maximization algorithm to cluster genotypes (Beugin et al. 2018) and runs much faster than STRUCTURE, which uses a Bayesian MCMC approach. Both rely on population genetic models assuming Hardy-Weinberg equilibrium (HWE) and linkage equilibrium within real clusters to calculate the likelihood of specific clustering solutions. DAPC is a model-free approach where the genotype data is first transformed using PCA, and then the principal components (PCs) are used as input for linear discriminant analysis (DA). As the three clustering methods yielded similar results, we only present the *snapclust* and STRUCTURE analyses here; details regarding the DAPC analysis can be found in the Supplementary (Analysis S1). For all clustering approaches, we arbitrarily assigned samples to a group if they showed a group membership probability >0.8.

We applied *snapclust* with default settings for numbers of genetic clusters (K) ranging from 1 to 20 and consulted the three information criteria AIC, BIC, and KIC to decide on the most probable K. The *snapclust* analysis suggested using a K value of 6 (see Results). However, the number of individuals assigned to the smallest cluster in this solution was more than 10× smaller than the number of individuals assigned to the largest cluster (Table S5), and uneven sample sizes can hamper the ‘correct’ identification of clusters (Kalinowski 2011; Neophytou 2014; Puechmaille 2016; Wang 2017). To break the influence the numerically dominant cluster might have on the detection of smaller clusters, we additionally ran *snapclust* on a more balanced subset of our data containing all samples from clusters 2–6 but only 222 samples from the largest cluster 1 (222 = mean number of samples in clusters 2–6). These samples consisted of the 15 *A. f. fabae* reference samples plus 207 samples selected randomly from those assigned to cluster 1 under K = 6.

We ran STRUCTURE with the admixture model and without prior information on sample origin. We used the settings suggested by Wang (2017) to improve detection of clusters in (possibly) unbalanced datasets. These settings include uncorrelated allele frequencies among populations (FREQSCORR = 0) and separate alpha values per population (POPALPHAS = 1, UNIFPRIORALPHA = 0), with an initial alpha of 0.17 (=1/6, six being the number of clusters inferred with *snapclust*). The other settings were left to their default. We ran ten independent simulations for each K between 1 and 10, doing 200,000 iterations after discarding the first 25,000 iterations as burn-in. We also ran STRUCTURE with the same settings on the more balanced data subset as described above. To infer the most probable number of genetic clusters we considered mean LnP(K) (Pritchard et al. 2000) and Evanno’s DeltaK (Evanno et al. 2005) as implemented in STRUCTURE HARVESTER (Earl and vonHoldt 2011). To summarize the output of the replicate STRUCTURE runs we used CLUMPAK (Kopelman et al. 2015).

### Genetic diversity, genetic differentiation, and host plant associations

To describe genetic diversity in the microsatellite dataset, we calculated the number of alleles, observed (H_o_) and expected (H_e_) heterozygosity for all microsatellite loci overall and for each of the six genetic groups inferred by STRUCTURE with *adegenet* 2.1.5 (Jombart 2008). We also tested for deviations from HWE overall and within the six groups using *pegas* 1.1 (Paradis 2010), and we calculated pairwise F_ST_ values (Weir and Cockerham 1984) between groups with the function *pairwise.WCfst* and 95% confidence intervals with *boot.ppfst* (nboots = 1000) using *hierfstat* 0.5–10 (Goudet 2005). To put these values in relation to genetic differentiation that may result from spatial or temporal separation, we further calculated pairwise F_ST_ values between the three sampling sites and the different sampling time points within each of the four dominant groups found on the winter host *E. europaeus* (clusters 1, 2, 3, and 5). Finally, we used Fisher’s exact tests with simulated *p*-values (number of simulations B = 2000) to test the null hypothesis of independence between the cluster to which a sample was assigned and the host plant from which it was collected. We separately tested for winter hosts and summer hosts, leaving out samples that were not assigned to any cluster according to STRUCTURE and the references samples from *A. f. fabae* and *A. f. cirsiiacanthoides*.

### Hybrid detection

To identify possible hybrids in a targeted manner we used the software *NewHybrids* 2.0 which applies a Bayesian clustering method (Anderson and Thompson 2002). The method requires the input data to consist of just two parental populations and their offspring. We therefore looked for hybrids separately in all pairwise combinations of the six genetic groups. Each of the input datasets consisted of the genotypes that were assigned to one of the two considered clusters with a probability >0.8, plus the genotypes whose assignment probabilities were highest to one and second highest to the other considered cluster, based on the STRUCTURE analysis under K = 6. We ran *NewHybrids* with a burn-in of 100,000 followed by 400,000 sweeps, using uniform priors for both π and θ, and looking for F1 hybrids only (to detect backcrosses, a larger number of markers than we have would be required). We considered those samples as hybrids that showed higher membership probability to the hybrid category than to either parental category. To estimate a detection probability for our approach, we applied *NewHybrids* to datasets containing simulated hybrids that we obtained with the R function *adegenet*::*hybridize*. As parental genotypes we used those individuals with an assignment probability >0.8 to the clusters under consideration in the STRUCTURE analysis and with no missing data. For each of the 15 combinations of parental clusters, we simulated 100 times 20 hybrids. On each dataset, we ran *NewHybrids* as above but with a burn-in of just 1000 followed by 4000 sweeps. The software detected 18.3 of the 20 simulated hybrids on average (91%, Table S13). However, the number of correctly detected simulated hybrids was particularly low for the two combinations of clusters 2 and 3 (10.3/20 = 52% of simulated hybrids detected) and 2 and 5 (16.1/20 = 80%) (Table S13). In summary, *NewHybrids* probably underestimates the number of hybrids for these cluster combinations, while the number of hybrids might be close to the actual number for the other combinations.

To test whether the frequency of putative hybrids differed between spring (March and April sampling timepoints) and autumn (late October sampling timepoint), we used Pearson’s χ^2^-square tests.

### Endosymbiont detection

In many herbivorous insect species and very prominently in the pea aphid, *Ac. pisum*, specialized host races are characterized by carrying differing complements of heritable endosymbionts (Ferrari et al. 2012). Since the endosymbionts provide various ecological functions to their hosts and are inherited from one generation to the next, differential endosymbiont prevalences among host taxa corroborate their ecological distinctiveness. In *A. fabae*, all aphid individuals carry the obligate endosymbiont *Buchnera aphidicola* (Douglas 1998). In addition, the facultative endosymbionts *Hamiltonella defensa* and *Regiella insecticola* occur frequently, while other known facultative endosymbionts are exceedingly rare (Gimmi et al. 2023). Therefore, to test whether also in *A. fabae* genetically differing groups show distinct endosymbiont prevalences, we determined the presence or absence of the obligate symbiont *B. aphidicola* (as a positive control) and of *H. defensa* and *R. insecticola* in all our aphid samples using diagnostic PCR. We did separate PCR reactions using specific primers for each endosymbiont and determined the presence or absence of amplified endosymbiont DNA using a QIAxcel capillary electrophoresis device. The PCR protocol and primer sequences are provided in Table S4. For the analysis, we filtered out samples with missing data (*A. f. fabae* and *A. f. cirsiiacanthoides* reference samples) or negative results for *B. aphidicola*, remaining with *N* = 2047 samples. We then tested for differences in the frequency of symbiotypes (Ham^**-**^Reg^**-**^, Ham^**-**^Reg^**+**^, Ham^**+**^Reg^**-**^ or Ham^**+**^Reg^**+**^) among genetic groups using pairwise Fisher’s exact tests and a Bonferroni-adjusted significance level.

## Results

### Genetic structure and host plant associations in the black bean aphid complex

When comparing observed heterozygosity (H_o_) with expected heterozygosity (H_e_) in our complete microsatellite dataset, we see the heterozygote deficit and significant deviation from Hardy-Weinberg equilibrium (HWE) expected for a dataset containing strong genetic structure (*p* < 0.0001 for all loci, Table S6).

Indeed, all clustering approaches we used point at there being multiple genetically divergent clusters in our dataset. Using *snapclust*, the information criteria suggest using a *K* value between 6 (BIC) and 7 or 8 (KIC and AIC, Fig. S1). Under K = 6, the reference samples get assigned to one cluster each and only 23 out of 2099 samples show a membership probability of less than 0.8 to any cluster. Under *K* = 7 or *K* = 8, many samples result as admixed between the new clusters, and the reference samples of *A. f. fabae* get assigned to two or three different clusters under *K* = 7 and *K* = 8, respectively (Fig. S3). Both these observations point at *K* = 6 being the best solution with *snapclust*. This is supported by all three information criteria suggesting *K* = 6 when *snapclust* is applied to the more balanced data subset (Fig. S2).

**Fig. 1:**
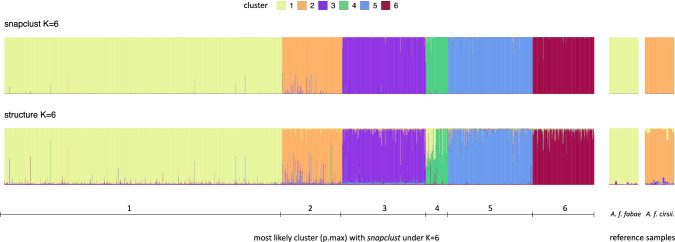
Clustering results under K = 6 from *snapclust* and STRUCTURE. Each aphid individual is represented by a vertical bar, the proportion of this bar in a specific color represents the likelihood that the sample belongs to the respective cluster (membership probability). For each K, the wide boxes to the left show all 2099 samples used in the analysis. The samples are ordered according to the cluster for which they show highest membership probability in the *snapclust* *K* = 6 result (x-axis). The two narrow boxes to the right zoom in on the reference samples known to represent *A. f. fabae* and *A. f. cirsiiacanthoides*, respectively.

While LnP(K) was not informative to infer K from the STRUCTURE output, Evanno’s DeltaK suggests *K* = 2 (Fig. S4), which in light of the *snapclust* results seems overly conservative. Running STRUCTURE on the more balanced dataset, there remains some uncertainty applying Evanno’s DeltaK due to inconsistent runs at *K* = 5, but the posterior probabilities clearly plateau at *K* = 6 (Fig. S5). We therefore decided to settle with *K* = 6 for the following analyses. The major difference between *snapclust* and STRUCTURE under *K* = 6 is the generally lower membership probabilities resulting from the latter, resulting in more samples that are not assigned to any cluster considering STRUCTURE (147 vs. 23 with *snapclust*, Fig. 1). Cluster assignment based on STRUCTURE is thus more restrictive, which is why we used it for the presented follow-up analyses.

**Fig. 2:**
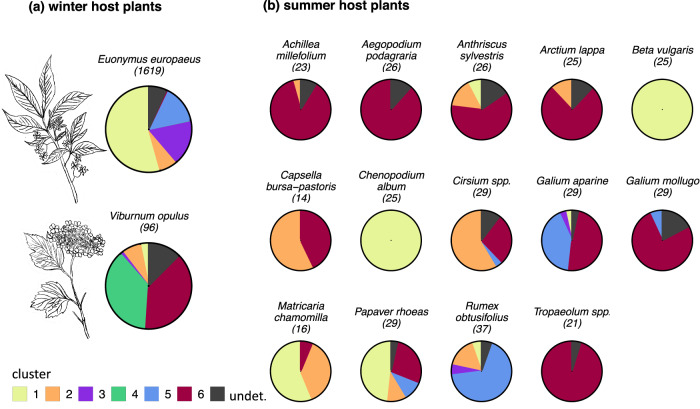
Relative frequency of aphid individuals assigned to the six genetic groups on the host plants considered for this study. Aphids were assigned to a cluster if they showed a membership probability >0.8 to it in the STRUCTURE results under *K* = 6. Samples that have a membership probability below 0.8 for all clusters are categorized as "undetermined." The number of aphid individuals considered per host plant is given in brackets. Each aphid individual was collected from a different plant individual. Note that the sample size is much larger for *E. euonymus* than for the other host plants. **a** Winter host plants; **b** summer host plants in alphabetical order.

Once the samples are clustered into six genetic groups, H_o_ and H_e_ are close to each other within the groups, and with the exceptions of one locus each in groups 1, 2, 4, and 6, there are no significant deviations from HWE (Table S6). There is a significant correlation between the genetic group to which aphid samples are assigned and the host plant from which they were collected, both considering winter hosts (*p* < 0.001 in Fisher’s exact test) and summer hosts (*p* < 0.001, Figs. 2, S6, Tables S7–S10).

Among the six genetic clusters, cluster 1 likely corresponds to *A. f. fabae*, as it contains the respective references samples and is the only cluster found associated with *A. f. fabae*’s diagnostic summer host plants *Beta vulgaris* and *Ch. album*. Samples assigned to cluster 1 were collected also from the summer hosts *An. sylvestris*, *G. aparine*, *M. chamomilla*, *P. rhoeas* and *R. obtusifolius* (Table S9). *A. f. fabae* was dominant on the winter host *E. europaeus* (880 of 1619 samples) but rare on the winter host *V. opulus* (3 of 96 samples, Table S7, Fig. 2).

Cluster 2 likely corresponds to *A. f. cirsiiacanthoides*, as it contains the respective reference samples and is the dominant cluster found associated with *Cirsium* spp., *A. f. cirsiiacanthoides*’ diagnostic summer hosts. It was also found on several other summer hosts, including *Ac. millefolium, An. sylvestris*, *Ar. lappa, Ca. bursa-pastoris*, *M. chamomilla*, *P. rhoeas*, and *R. obtusifolius*. Aphids belonging to cluster 2 occurred on both winter hosts in similar frequencies (7% of all samples for both, Tables S7 and S9).

Cluster 6 likely corresponds to *A. f. mordwilkoi*, as it was dominant on this subspecies’ diagnostic summer hosts *Ar. lappa* and *T. majus*. Cluster 6 was also dominant on *Ac. millefolium*, *Ae. podagraria*, *An. sylvestris*, *G. aparine* and *G. mollugo*, and single individuals were collected from *C. bursa-pastoris*, *Cirsium* spp., *M. chamomilla*, and *P. rhoeas*. *A. f. mordwilkoi* was almost absent from *E. europaeus* (6 of 1619 samples) but frequent on *V. opulus* (37 of 96).

The remaining three clusters are less straightforward to identify, and we refer to the Discussion for their possible assignment to known taxa. Both cluster 5 and cluster 3 seem to use only *E. europaeus* as winter host (Fig. 2, Table S7). In summer, we collected aphids assigned to cluster 5 mostly from *G. aparine* and *R. obtusifolius*, while cluster 3 was virtually absent from the summer hosts we sampled (one sample on *G. aparine* and two on *R. obtusifolius*, Table S9, Fig. 2). Cluster 4 was mostly collected from *V. opulus* as primary host (36/38 samples) and was not observed on any of the sampled secondary host plants.

**Fig. 3:**
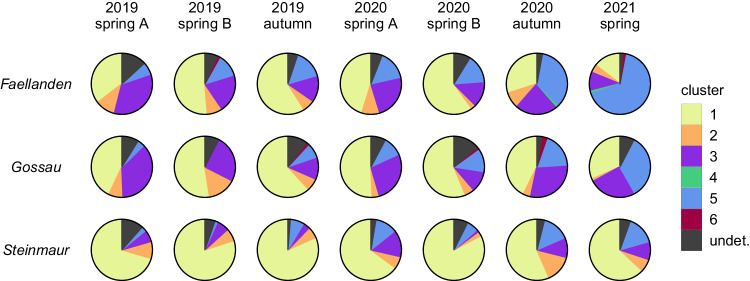
Distribution of individuals belonging to the six genetic clusters defined by STRUCTURE on the winter host *E. euonymus* between sites (*Faellanden*, *Gossau*, and *Steinmaur*) and over time. The four main genetic clusters (yellow, orange, violet, and blue) are present at all sites at all but one time point.

The pairwise F_ST_ values between the six genetic clusters are all significantly larger than zero, but the extent of genetic differentiation varies (Table 1). *A. f. fabae* and cluster 4 are most strongly differentiated from all other clusters with pairwise F_ST_ values ranging from 0.094 to 0.128 and 0.093 to 0.128, respectively. The remaining four groups are more closely related to each other with pairwise F_ST_ values between 0.050 and 0.070 (Table 1). The large number of samples collected from *E. europaeus* at three distinct sites and at different time points allow us to put these values in relation to genetic differentiation that may result from spatial or temporal separation. The relative proportions of the four genetic clusters dominating on *E. europaeus* showed some variation across space and time (Fig. 3), but within these groups, genetic differentiation was very weak, with F_ST_ values between sites or between time points vastly smaller than those between genetic clusters, and with confidence intervals that included zero in the majority of comparisons (Table S11, S12).

**Table 1:** Pairwise F_ST_ values (Weir and Cockerham) and 95% confidence intervals (values in brackets) between the six main genetic groups identified in our dataset, considering the clustering solution from STRUCTURE under *K* = 6 and assigning samples to a cluster if they show an assignment probability >0.8.

	2 - orange	3 - violet	4 - green	5 - blue	6 - red
**1 – yellow** *A. f. fabae*	0.094 [0.062, 0.127]	0.104 [0.072, 0.138]	0.128 [0.088, 0.17]	0.128 [0.100, 0.155]	0.105 [0.072, 0.139]
**2 - orange** *A. f. cirsii*.		0.050 [0.032, 0.069]	0.095 [0.058, 0.133]	0.053 [0.033, 0.077]	0.054 [0.032, 0.078]
**3 - violet**			0.093 [0.050, 0.141]	0.068 [0.046, 0.087]	0.063 [0.041, 0.085]
**4 - green**				0.120 [0.082, 0.159]	0.104 [0.071, 0.136]
**5 - blue**					0.070 [0.048, 0.095]

### Evidence for hybridization between taxa

73 samples were inferred to be putative hybrids (Table S14, S15); 71 of them were among the 143 samples not assigned to any cluster in the main STRUCTURE analysis (‘undetermined’ samples). The putative hybrids show to the largest part admixture between clusters 1 and 4 (37 samples) and between clusters 2 and 5 (20 samples). The 1 × 4 hybrids have all been collected in spring over all sampling years and from both *E. europaeus* and *V. opulus* (32/1165 = 3% of *E. europaeus* spring samples, 5/91 = 5% of *V. opulus* spring samples). As expected for hybrid genotypes, H_o_ (0.80) is distinctly larger than H_e_ (0.66) within these putative 1 × 4 hybrids, and allele distributions are intermediate between those of cluster 1 and cluster 4 (Fig. S7). The putative 2 × 5 hybrids have all been collected from *E. europaeus* spread over all three sampling years, 18 samples in March and April and two in October. For these putative hybrids, allele distributions are intermediate between those of cluster 2 and cluster 5 (Fig. S8), but there is hardly any difference between H_o_ (0.70) and H_e_ (0.69). For all other pairs of clusters, we found between zero and three putative hybrids (Tables S14 and S15). Overall, 67 putative hybrids were collected in spring months (5% of spring samples from the winter hosts *E. europaeus* and *V. opulus*) and two in autumn (0.4% of autumn samples from *E. europaeus*). Hybrids were thus more frequent in spring than in autumn (χ^2^ = 19.6, df = 1, *p* < 0.0001). The remaining four hybrids were collected from summer host plants, one each from *An. sylvestris* and *Ci. vulgare* (hybrids of clusters 2 and 6), *T. majus* and *P*. *rhoeas* (hybrids of clusters 5 and 6).

### Endosymbiont prevalence in *Aphis fabae* genetic clusters

**Fig. 4:**
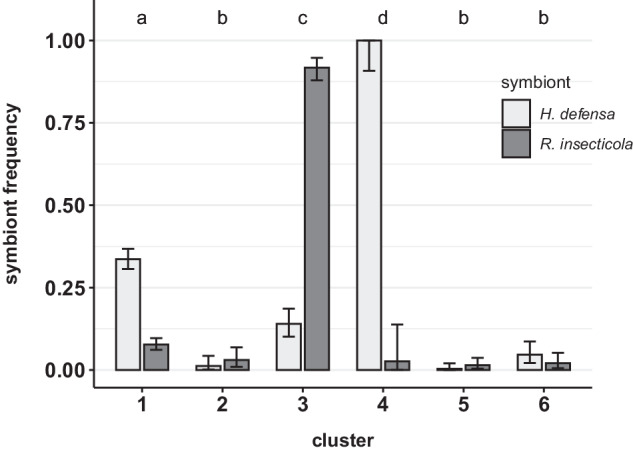
Frequencies of the two endosymbiotic bacteria species *Hamiltonella defensa* and *Regiella insecticola* in each of the six genetic clusters of *A. fabae*, assigning samples based on the STRUCTURE results. Different letters indicate significant differences in symbiotypes in pairwise comparisons using Fisher’s exact tests with Bonferroni corrections (see Table S17 for *p*-values). The error bars indicate binomial proportion confidence intervals.

The genetic clusters we identified with microsatellite genotypes exhibit significant differences in the prevalence of the two endosymbionts *Hamiltonella defensa* and *Regiella insecticola* (Fig. 4, Tables S16, S17). In cluster 1 (*A. f. fabae*) we found *H. defensa* in 34% and *R. insecticola* in 8% of the aphids. Cluster 3 shows a lower *H. defensa* (14%) and a much higher *R. insecticola* frequency (92%), while in cluster 4, 100% of the aphids carried *H. defensa* but only 3% *R. insecticola*. In the remaining three clusters, both endosymbionts are very rare (Fig. 4, Table S16). Accordingly, the symbiotypes of clusters 2, 5, and 6 do not significantly differ from each other, but they all differ from the three groups with higher endosymbiont prevalence (Fig. 4, Table S17).

Among the 37 putative hybrids between clusters 1 and 4, 41% carry *H. defensa* and 5% *R. insecticola*. Statistically, symbiotypes prevalence in the 1 × 4 hybrids was not different from the prevalence in cluster 1 (*p* = 0.780 in Fisher’s Exact test) but different from the prevalence in cluster 4 (*p* < 0.000 in Fisher’s Exact test). Among the putative 2 × 5 hybrids, no sample carried *H. defensa* and one *R. insecticola*, which does not differ from the findings for cluster 2 or 5 (Tables S16, S17).

## Discussion

Different plant species may impose divergent selection on the phytophagous insects exploiting them, but for the evolution and maintenance of genetically differentiated host races, reduced gene flow between host-associated populations is necessary. Here we show that despite a shared mating habitat, black bean aphids of the *A. fabae* complex can be assigned to at least six genetically distinct groups, which differ in their host associations and in the frequency of infection with facultative endosymbionts.

It is uncontested that such ecological diversification in phytophagous insects is facilitated when the same host plant represents adult and larval food source as well as mating site (Bush 1975; Caillaud and Via 2000; Drès and Mallet 2002; Feder et al. 1994), but our example shows that such tight linkage is not a strict requirement. A similar argument has been made for the Lepidoptera (butterflies and moths), where there is ample evidence for larval host plant-associated diversification (Braby and Trueman 2006; Braga et al. 2018; Fordyce 2010), even though adults use different food sources (often nectar) and typically mate off the larval food plants, sometimes even forming mating aggregations on hilltops (e.g. Prieto and Dahners 2009). It is noteworthy, though, that a link between adult and larval food sources may still exist in the Lepidoptera, since nectar-feeding adults include the larval food plant in their diet more often than expected by chance (Altermatt and Pearse 2011). In the *A. fabae* complex, a permanent link of adult and larval feeding sites with mating sites exists only for those taxa that are not host-alternating.

The existence of multiple *A. fabae* taxa characterized by distinct feeding preferences has been described already 100 years ago (Börner and Janisch 1922). However, the assignment of individuals to taxa using host plant choice tests may hinge on the developmental stage and the condition of both host plants and aphids (Thieme 1987), as well as on the degree of phenotypic plasticity in aphid performance traits (Gorur et al. 2005; Gorur et al. 2007). Still, in accordance with and extending on previous population genetic studies (Jörg and Lampel 1996; Vorburger et al. 2017), our data shows that there is clear genetic differentiation between black bean aphids that dominate on plants considered ’diagnostic,’ and thus between ecologically defined taxa. This attests to the careful work of the entomologists who studied this complex group with biological assays. It is particularly interesting considering the lack of resolution in mitochondrial COI/II and CytB sequences (Béji et al. 2015; Coeur d’acier et al. 2007; Coeur d’acier et al. 2014; Zhang et al. 2010), which suggests that the *A. fabae* lineages represent evolutionarily young taxa.

Most authors agree that the winter host *E. europaeus* is principally used by four *A. fabae* taxa (summarized by Blackman and Eastop 2017, see also Table S1). This matches well with the four main genetic groups (1, 2, 3, and 5) we found on *E. europaeus*, two of which we can clearly identify as *A. f. fabae* and *A. f. cirsiiacanthoides*. The other two taxa expected on *E. europaeus* are *A. solanella*, whose diagnostic summer host is *Solanum nigrum*, and *A. evonymi*, which is monoecious and feeds on *E. europaeus* throughout the season. As we do not have samples from either diagnostic summer host, we cannot unambiguously assign the clusters we found to the two taxa. However, we propose that cluster 3 might be either *A. solanella* or *A. evonymi* (we found samples assigned to cluster 3 almost exclusively on *E. europaeus*), while cluster 5 might be *A. solanella* but not *A. evonymi*, as we found it in relevant numbers on the (non-diagnostic) summer host plant *R. obtusifolius* and also on *Galium* species and *P. rhoeas* (Fig. 2, Table S9). Two arguments might challenge these hypotheses: first, *A. evonymi* is generally thought to differ visually from other *A. fabae* taxa due to its brownish body coloration (Blackman and Eastop 2000), but no such divergent body color was recorded during sample collection (E. Gimmi: personal observation). Second, both *A. evonymi* and *A. solanella* are considered independent species, which stands in contrast to our finding that clusters 3 and 5 are closely related to each other and to *A. f. cirsiiacanthoides* and *A. f. mordwilkoi* (Table 1). Nevertheless, because coloring is variable and the taxonomy not fully resolved, we suppose that cluster 3 is *A. evonymi*, and cluster 5 is *A. solanella*. Considering F_ST_ values in isolation, one could argue that cluster 1, cluster 4, and the clusters 2, 3, 5, and 6 together correspond to three different species or taxa, while clusters 2, 3, 5, and 6 correspond to subspecies or subtaxa (Table 1). A larger number of genetic markers, if not a genome-wide sequencing, would be useful to test this hypothesis.

On *V. opulus*, we could confirm the presence of two taxa expected to use this shrub as primary host according to the literature: *A. f. mordwilkoi* (cluster 6 – identified by its summer host associations) and *A. f. cirsiiacanthoides*. We did not expect to find yet another very abundant cluster that appears to be *V. opulus*-specific (cluster 4, green). It could either represent a yet undocumented *A. fabae* host race, or a different but closely related aphid species that we mistook for *A. fabae* when identifying aphids only by the unaided eye in the field. With hindsight, we suspect cluster 4 to represent *A. viburni*, a monoecious taxon that feeds on *V. opulus* throughout the year and is generally regarded as a member of the *A. fabae* complex in the broad sense (Blackman and Eastop 2000). *A. viburni* would show morphological differences to other black bean aphids under microscopic examination (Lampel and Meier 2007), but since we extracted DNA destructively for this study, we would need to collect new aphid samples to confirm our hypothesis. According to Coeur d’acier et al. (2007; 2014), mitochondrial markers cannot distinguish between *A. viburni* and members of *A. fabae* s. str. This is compatible with our finding that the nuclear genetic differentiation of cluster 4 from other clusters is comparable to that of *A. f. fabae* (Table 1) also under the assumption that cluster 4 corresponds to *A. viburni*.

We here confirm that there is genetic differentiation and thus restricted genetic exchange between black bean aphids associated with different secondary host plants. The relatively low number of hybrids we found is additionally indicating the presence of prezygotic or early-life postzygotic barriers to gene flow. Different mechanisms might play a role: one possibility is a difference in the timing of arrival and the production of sexual morphs on the primary host plant. Such temporal separation plays an important role in the maintenance of genetically divergent lineages in another host alternating aphid, *Rhopalosiphum padi* (Halkett et al. 2006; Halkett et al. 2005). While we cannot exclude some variation in the timing of sexual reproduction, this mechanism is unlikely to be relevant for reproductively separating the four main *A. fabae* groups on *E. europaeus*, since all of them were present simultaneously on *E. europaeus* in autumn of both sampling years (Fig. 3). However, separate temporal niches might be realized at a smaller scale, for example can mating-related activities of different taxa be unequally distributed over the day (Thieme and Dixon 1996). Also, behavioral mechanisms may contribute to reproductive isolation between *A. fabae* taxa: there is for instance evidence that male black bean aphids are able to differentiate between female pheromones of their own and of different taxa (Raymond et al. 2001; Thieme and Dixon 1996). We could also imagine that a behavioral preference for chemical signals from the summer host plants of specialized taxa could promote assortative mating and thus reduce gene flow between lineages. Which of these mechanisms is actually relevant for reproductive isolation among taxa within the *A. fabae* complex remains to be tested.

Assortative mating is selected for when hybrid offspring show reduced fitness. Based on a number of crossing experiments (Iglisch 1968; Raymond et al. 2001; Thieme 1988; Tosh et al. 2004), we can assume that reproductive success might be lower for mixed-taxa pairs than for same-taxa ones, but that viable and fertile hybrid offspring are possible. For example, Raymond et al. (2001) found hybrids between *A. f. fabae* and *A. f. mordwilkoi* to be viable but to produce fewer eggs (less than a third) than pure-bred offspring from either parental taxa. In agreement with that, we identified certain individuals in our dataset as putative hybrids (Tables S14, S15). The fact that most of these putative hybrids were collected in spring rather than in autumn is suggestive for selection acting against hybrids during the summer months, thereby reinforcing reproductive isolation between genetic groups (Howard 1993). Postzygotic selection may have an intrinsic (e.g. genetic incompatibility of parental chromosomes) or extrinsic basis, and the latter can directly be related to ecological speciation models: extrinsic postzygotic selection may manifest specifically in the environments that parental individuals are adapted to if the intermediate allele composition present in hybrids results in a reduced fitness compared to adapted parents (‘maladaptive intermediacy’, Hatfield and Schluter 1999; Rundle and Whitlock 2001). While it is not possible from our observational data to distinguish between intrinsic and extrinsic selection against hybrids (Rundle and Whitlock 2001), clear evidence for extrinsic postzygotic selection has been shown for other phytophagous insect systems (Funk 2010; Nosil et al. 2003) and might be tested for specifically in *A. fabae* in future experiments.

While hybridization between taxa co-occurring on the same winter hosts could be expected, we were surprised by the relatively large number of putative hybrids between cluster 1 (*A. f. fabae*), using *E. europaeus* as primary host, and cluster 4 (presumed *A. viburni*), using *V. opulus* as primary host. We observed these hybrids only in spring and on both primary host plants. That these taxa are reproductively compatible is consistent with experimental evidence from Iglisch (1968). But how are hybrids formed when the parental taxa mate on different hosts? We hypothesize that male aphids (which we did not sample) occasionally visit the ‘wrong’ hosts when actively searching for females during the period of sexual reproduction. This would be a straightforward explanation for the winged males of *A. f. fabae*, but less so for males of *A. viburni*, which are reported to be unwinged (Heie 1986). However, *E. europaeus* and *V. opulus* are very common hedgerow plants in our sampling area, often growing with intertwined branches. It would therefore at least be feasible that stray males of either taxon could mate with egg-laying females that are already settled on the correct plant species. This might explain the presence of hybrids on both winter hosts in spring despite the strict host specificity observed for the female aphids. The vicinity of the two winter host plants might also explain why genetic differentiation among taxa using the same winter host is not different from genetic differentiation among taxa using different winter hosts (cf. Table 1).

The existence of hybrids between cluster 2 (*A. f. cirsiiacanthoides*) and cluster 5 is less surprising, as they both mate on *E. europaeus*. The comparably high number (Table S14) might be either cause or result of these clusters being little differentiated (Table 1), though for the similarly differentiated combination of clusters 2 × 3 and 2 × 6, we found just one and two hybrids, respectively (Table S14, S15). Interestingly, the two 2 × 6 hybrids were both collected from summer hosts that are used by both parental clusters (*Cirsium* spp. and *An. sylvestris*). Based on this anecdotal observation, a future experiment might test whether hybrid fitness is different on host plants that are used by both parents compared to host plants that are used by either parent. This might help us understand to what extent reduced hybrid fitness is based on extrinsic compared to intrinsic fitness effects.

The correlation between the use of specific host plants and genetic differentiation in black bean aphids, combined with performance trade-offs on these different plants (Douglas 1997; Mackenzie 1996; Müller 1982), recapitulate the situation of host specialized biotypes in the pea aphid (Peccoud et al. 2009; Via 1999). The differential prevalence of heritable endosymbiotic bacteria in host-associated taxa represents another parallel between the two systems (Ferrari et al. 2012; Simon et al. 2003). In *A. fabae*, the prevalences of the two heritable facultative symbionts *H. defensa* and *R. insecticola* differ markedly among the different genetic groups (Fig. 4). These frequency differences may have arisen due to drift and could thus just be a consequence of the reproductive barriers existing between taxa. However, *H. defensa* and *R. insecticola* may provide their host with various ecological benefits including protection against parasitoids or pathogens (reviewed in Guo et al. 2017), but they also entail fitness costs (Polin et al. 2014; Vorburger and Gouskov 2011; Zytynska et al. 2021). Net costs are known to vary depending on the aphid’s host plant environment (McLean et al. 2011; Sochard et al. 2019). It is therefore likely that differing costs and benefits of hosting heritable endosymbionts, and thus diverging selection, account for the large differences in symbiont prevalence between *A. fabae* taxa specialized on different plant species (Fig. 4). Some symbionts can even directly affect aphid performance on certain host plants (Tsuchida et al. 2004; Wagner et al. 2015). No such effect is known yet for *H. defensa* or *R. insecticola*, but this might be worth to investigate in a future experiment. In either case, the symbiont frequency differences represent additional evidence for divergent ecological selection on different host plants.

In conclusion, we illustrate an example of genetic divergence within a species complex of host-alternating aphids that correlates with the association with different host plants. Genetic divergence is also correlating with differences in the frequency of infection with facultative endosymbionts. Both is suggestive of divergent selection underlying the observed differentiation, similar to host-associated diversification in other phytophagous insects. The advantage of ecological specialization seems to be strong enough to promote the maintenance of genetic divergence despite the opportunity for gene flow at shared mating sites, and this is likely achieved via an interplay of prezygotic barriers and postzygotic selection against hybrids.

### Supplementary information

Supplementary Material

## Data Availability

Data and scripts generated in this study are available at Dryad Digital Repository: 10.5061/dryad.tx95x6b5t.
