# Supplementary material for: Ecological divergence despite common mating sites: Genotypes and symbiotypes shed light on cryptic diversity in the black bean aphid species complex
Source: Heredity (Edinb). 2024 May 14;132(6):320–30. doi: 10.1038/s41437-024-00687-0 (PMC11167045; doi:10.1038/s41437-024-00687-0)
Supplement: Supplementary file 1 — Supplementary Material [file 41437_2024_687_MOESM1_ESM.pdf]

Supplementary Material

for manuscript '*Ecological divergence despite common mating sites: Genotypes and symbiotypes shed light on cryptic diversity in the black bean aphid species complex*'

submitted to Heredity, March 2024

authors: Elena Gimmi, Jesper Wallisch & Christoph Vorburger

Index

Supplementary Tables S1-S17 2

Supplementary Figures S1-S8 19

Supplementary Analysis: genetic clustering using DAPC 26
    Supplementary Figures S9-S11 27

References 29

Table S1: A: The five members of the *Aphis fabae* group according to Blackman and Eastop (2017) and their main host plants. B: Four additional aphid species that are currently counted towards the *Aphis fabae* group sensu largo and that might be found on the same hosts as *Aphis fabae*. As far as known, they are morphologically distinct from member of the *Aphis fabae* group sensu stricto but genetically weakly resolved. Summarised from the following online databases: http://www.aphidsonworldsplants.info (accessed 25.08.2023, Blackman and Eastop (2000)), https://influentialpoints.com (accessed 25.08.2023, B. Dransfield and B. Brightwell), http://aphid.speciesfile.org (accessed 24.09.2023, C. Favret and D. Eades).

	**current name**	**synonyms (selection)**	**main winter hosts**	**diagnostic summer hosts**	**other summer hosts (selection)**	**notes**
**A**	*Aphis fabae fabae*	*Aphis fabae* sensu stricto	*Euonymus europaeus*	*Vicia faba*	*Beta vulgaris, Chenopodium spp., Matricaria spp., Papaver spp., Rumex obtusifolius*	dioecious
	Aphis fabae cirsiiacanthoidis	*Aphis acanthi, possibly Aphis janischi*	*Euonymus europaeus, Viburnum opulus, Philadelphius coronarius*	*Cirsium spp.*	*Rumex obtusifolius*	dioecious
	Aphis fabae mordwilkoi	*Aphis barberae*	*Viburnum opulus, Philadelphius coronarius*	*Tropaeolum spp., Arctium spp.*	*Rumex obtusifolius*	dioecious
	Aphis solanella		*Euonymus europaeus, (Viburnum opulus, Philadelphius coronarius)*	*Solanum nigrum.*	*Rumex obtusifolius*	dioecious; may survive on *V. opulus* and *P. coronarius* but worse than on *E. europaeus*.
	Aphis evonymi	*Aphis cognatella*	*Euonymus europaeus*	*Euonymus europaeus*		monoecious; brownish body colour
**B**	*Aphis viburni*	possibly *A. epipactis,* possibly *Aphis illicis*	*Viburnum opulus*	*Viburnum opulus*		monoecious on *Viburnum*; brown-greenish-blackish body colour; "long-haired" compared to *A. fabae* s. str.
	Aphis epipactis		*(Viburnum opulus)*	*Epipactis spp.*		monoecious on *Epipactis*
	Aphis ilicis		*(Viburnum opulus)*	*Ilex aquifolium*		monoecious on *Ilex*
	Aphis hederae		*(Viburnum opulus)*	*Hedera helix*		monoecious on *Hedera*

Table S2: Sampling data overview: number of black bean aphid samples that are included in the presented dataset, per sampling time point, sampling site and host plant species.

Host plant species	sampling time point	site	N
Euonymus europaeus	Mar.19	Faellanden	76
Euonymus europaeus	Mar.19	Gossau	79
Euonymus europaeus	Mar.19	Steinmaur	78
Euonymus europaeus	Apr.19	Faellanden	74
Euonymus europaeus	Apr.19	Gossau	80
Euonymus europaeus	Apr.19	Steinmaur	75
Euonymus europaeus	Oct.19	Faellanden	78
Euonymus europaeus	Oct.19	Gossau	76
Euonymus europaeus	Oct.19	Steinmaur	82
Euonymus europaeus	Mar.20	Faellanden	84
Euonymus europaeus	Mar.20	Gossau	77
Euonymus europaeus	Mar.20	Steinmaur	77
Euonymus europaeus	Apr.20	Faellanden	80
Euonymus europaeus	Apr.20	Gossau	80
Euonymus europaeus	Apr.20	Steinmaur	81
Viburnum opulus	Apr.20	Faellanden	15
Viburnum opulus	Apr.20	Steinmaur	19
Euonymus europaeus	Oct.20	Faellanden	70
Euonymus europaeus	Oct.20	Gossau	75
Euonymus europaeus	Oct.20	Steinmaur	76
Euonymus europaeus	Apr.21	Faellanden	69
Euonymus europaeus	Apr.21	Gossau	79
Euonymus europaeus	Apr.21	Steinmaur	73
Viburnum opulus	Apr.21	Faellanden	19
Viburnum opulus	Apr.21	Gossau	17
Viburnum opulus	Apr.21	Steinmaur	21
Viburnum opulus	summer 21	Zurich region	5
Achillea millefolium	summer 21	Zurich region	23
Aegopodium podagraria	summer 21	Zurich region	26
Anthriscus sylvestris	summer 21	Zurich region	26
Arctium lappa	summer 21	Zurich region	25
Beta vulgaris	summer 21	Zurich region	25
Capsella bursa-pastoris	summer 21	Zurich region	14
Chenopodium album	summer 21	Zurich region	25
Cirsium arvense & C. vulgare	summer 21	Zurich region	29
Galium aparine	summer 21	Zurich region	29
Galium mollugo	summer 21	Zurich region	29
Matricaria chamomilla	summer 21	Zurich region	16
Papaver rhoeas	summer 21	Zurich region	29
Rumex spp	summer 21	Zurich region	37
Tropaeolum majus	summer 21	Zurich region	21
Chenopodium album	samples from Vorburger *et al.* 2017		15
Cirsium arvense & C. vulgare	samples from Vorburger *et al.* 2017		15

Table S3: Cycling conditions and primers for used for microsatellite PCR; as applied for and presented in the manuscript "Defensive symbiosis in the wild: seasonal dynamics of parasitism risk and symbiont-conferred resistance" (Gimmi *et al.*, 2023). The primers were published by Coeur d'acier *et al.* (2004).

**Marker**	**size range [bp]**	**primer name**	**sequence**
AfF	113 – 204	AfF forward	GCGTTGCAGCAGCATATACT
		AfF reverse	CCTATATCGTGTGCGTGCAT
Af82	159 – 236	Af82 forward	GCGTAATGCAAGTAACGACC
		Af82 reverse	CGTCGTTCCAGCGAATTCTC
Af86	207 – 221	Af86 forward	CGCGTTCTCTCCAATAACTC
		Af86 reverse	TAATGTTGCGGATTGTTTGC
Af85	208 – 228	Af85 forward	CGCGTGCAGTGTAGGTCCAT
		Af85 reverse	CAAGGTGCGATTGACGACGA
Af50	255 – 276	Af50 forward	TGGTGAGTGCAGGCTAGTAT
		Af50 reverse	AAGGCACTTAGTCGACGTGT
Afbeta	260 – 377	Afbeta forward	GAGGACGCGGCTAAGAAGAA
		Afbeta reverse	CGAAAAGGGACGTCTACGAG
Af48	303 - 355	Af48 forward	TTAAACCTTTGAGCGTAGCG
		Af48 reverse	CCGAAGCAGCAGTAACATTG
Af181	299 - 362	Af181 forward	GGCATGTGCACGACGAATAC
		Af181 reverse	CGTTTCTTCGTGTGCGATTT

PCR PROTOCOL

**temp [°C]**	**time [min]**	**cycles**
95	15	
94	0.5	
60	1.5	30 x
72	1	
60	30	

PCR REACTION MIX (per sample)

**Reagent**	**volume [µl]**
ddH20 + Primers (conc. See table on the right)	4.5
QIAGEN Multiplex PCR Master Mix	5.5
DNA solution	1
Final vol per reaction	11

**Primer and label**	**conc. in PCR [µM]**
AfF forward + PET	0.1
AfF reverse	0.1
Af82 forward + NED	0.4
Af82 reverse	0.4
Af86 forward + VIC	0.2
Af86 reverse	0.2
Af85 forward + FAM	0.2
Af85 reverse	0.2
Af50 forward + PET	0.2
Af50 reverse	0.2
Afbeta forward + NED	0.4
Afbeta reverse	0.4
Af48 forward + VIC	0.4
Af48 reverse	0.4
Af181 forward + FAM	0.2
Af181 reverse	0.2

Table S4: Cycling conditions and primers used for symbiont-diagnostic PCR.

PRIMERS SYMBIONT DIAGNOSIS

symbiont	product size [bp]	primer F	sequence Primer F	primer R	sequence Primer R	reference
Buchnera aphidicola	196	16SA1	AGAGTTTGATCMTGGCTCAG	Buch_R_CV2	CCCCCACTTTRGTTTTTCAAC	Hafer-Hahmann and Vorburger (2020)
Hamiltonella defensa	471	10F	AGTTTGATCATGGCTCAGATTG	T419R	AAATGGTATTCGCATTTATCG	Ferrari *et al.* (2012)
Regiella insecticola	480	10F	AGTTTGATCATGGCTCAGATTG	U443R	GGTAACGTCAATCGATAAGCA	Ferrari *et al.* (2012)

PCR REACTION MIX (per sample)

reagent	volume [µl]
ddH20	2.3
Promega GoTaq® G2 Colorless Master Mix	5.5
Primer F	1.1
Primer R	1.1
Reagent mix per reaction	10
DNA solution per reaction	1
Final vol per reaction	11

PCR PROTOCOL

temp [°C]	time [min]	cycles
95	3	
95	0.5	
65-56	0.5	10x
72	1	
95	0.5	
55	0.5	25x
72	1	
72	6	

Table S5: Number of samples assigned to each cluster with *snapclust* or STRUCTURE under K=6, using an assignment threshold of p>0.8 (p is the group membership probability).

	**1 (yellow)** *snapclust*	**2 (orange)** *snapclust*	**3 (violet)** *snapclust*	**4 (green)** *snapclust*	**5 (blue)** *snapclust*	**6 (red)** *snapclust*	**undet.** *snapclust*	**samples per cluster STRUCTURE**
**1 (yellow) STRUCTURE**	976	0	0	0	0	0	0	**976**
**2 (orange) STRUCTURE**	0	181	0	0	0	0	0	**181**
**3 (violet) STRUCTURE**	0	0	280	0	0	0	0	**280**
**4 (green) STRUCTURE**	0	0	0	38	0	0	0	**38**
**5 (blue) STRUCTURE**	0	0	0	0	277	0	0	**277**
**6 (red) STRUCTURE**	0	0	0	0	0	200	0	**200**
**undet. STRUCTURE**	9	22	15	40	20	18	23	**147**
**samples per cluster** *snapclust*	**985**	**203**	**295**	**78**	**297**	**218**	**23**	**2099**

Table S6: Summary of allele numbers, observed and expected heterozygosity, and p-value of an exact test for HWE per locus; for the full dataset and for each of the six genetic groups inferred with STRUCTURE. P-values from HWE tests <0.05 are printed in bold, p-values below the Bonferroni-corrected significance threshold of 0.05/64=0.0008 are printed in red.

	alleles / locus	Ho	He	exact HWE
**full data (N=2099, 1.37% missing data)**				
Af85	11	0.56	0.67	**0.0000**
Af181	33	0.7	0.81	**0.0000**
Af86	7	0.25	0.57	**0.0000**
Af48	23	0.69	0.85	**0.0000**
Af82	38	0.66	0.86	**0.0000**
Afbeta	58	0.67	0.83	**0.0000**
AfF	44	0.69	0.84	**0.0000**
Af50	7	0.56	0.76	**0.0000**
mean	27.63	0.6	0.77	
total	221			
**group 1 (N=976, 1.29% missing data)**				
Af85	6	0.55	0.57	**0.0410**
Af181	6	0.64	0.67	0.3228
Af86	6	0.44	0.5	**0.0000**
Af48	11	0.75	0.77	0.1729
Af82	17	0.49	0.49	0.8159
Afbeta	22	0.57	0.59	0.4306
AfF	13	0.56	0.57	0.3098
Af50	5	0.61	0.6	0.3633
mean	10.75	0.58	0.6	
total	86			
**group 2 (N=181, 1.66% missing data)**				
Af85	6	0.56	0.61	**0.0013**
Af181	18	0.83	0.87	0.0907
Af86	2	0.01	0.01	1.0000
Af48	12	0.66	0.71	0.1488
Af82	21	0.85	0.89	0.1065
Afbeta	38	0.84	0.92	**0.0046**
AfF	33	0.68	0.89	**0.0000**
Af50	5	0.49	0.61	**0.0056**
mean	16.88	0.62	0.69	
total	135			

Table continues on the next side

Continuation Table S5

	alleles / locus	Ho	He	exact HWE
**group 3 (N= 280, 2.19% missing data)**				
Af85	5	0.5	0.51	0.3277
Af181	17	0.5	0.53	**0.0061**
Af86	3	0.1	0.09	1.0000
Af48	13	0.76	0.8	0.1385
Af82	15	0.83	0.8	0.6533
Afbeta	46	0.89	0.94	0.0904
AfF	21	0.81	0.83	0.4031
Af50	4	0.4	0.43	0.3337
mean	15.5	0.6	0.62	
total	124			
**group 4 (N=38, 0.99% missing data)**				
Af85	3	0.24	0.22	1.0000
Af181	7	0.68	0.73	0.1947
Af86	2	0.03	0.03	1.0000
Af48	7	0.69	0.54	0.6671
Af82	3	0.55	0.49	0.6466
Afbeta	10	0.47	0.59	**0.0007**
AfF	13	0.89	0.86	0.1945
Af50	4	0.35	0.3	1.0000
mean	6.13	0.49	0.47	
total	49			
**group 5 (N=277, 1.08% missing data)**				
Af85	4	0.57	0.54	0.7018
Af181	23	0.84	0.85	0.6443
Af86	3	0.06	0.06	1.0000
Af48	11	0.32	0.3	0.6551
Af82	21	0.79	0.8	0.1870
Afbeta	26	0.7	0.71	0.2987
AfF	25	0.84	0.86	0.1397
Af50	4	0.56	0.54	0.1600
mean	14.63	0.59	0.58	
total	117			
**group 6 (N=200, 0.88% missing data)**				
Af85	9	0.7	0.72	0.5234
Af181	13	0.79	0.81	0.8583
Af86	3	0.04	0.12	**0.0000**
Af48	13	0.77	0.78	0.3573
Af82	25	0.75	0.83	**0.0166**
Afbeta	22	0.72	0.76	0.4838
AfF	12	0.8	0.84	0.4636
Af50	6	0.54	0.57	**0.0416**
mean	12.88	0.64	0.68	
total	103			

Table S7: Contingency table showing the number of samples per genetic group per winter host (assignments based on **STRUCTURE** under K=6). A Fisher's Exact Test (simulated p-value based on 2000 replicates) on a version of this table with the row for undetermined samples removed (these are the samples that are not assigned to any cluster with p>0.8) results in a p-value <0.001.

group	*E. europaeus*	*V. opulus*	total
1	880	3	883
2	112	7	119
3	276	1	277
4	2	36	38
5	234	0	234
6	6	37	43
undet.	109	12	121
total	1619	96	1715

Table S8: Contingency table showing the number of samples per genetic group per winter host (assignments based on ***snapclust*** under K=6). A Fisher's Exact Test (simulated p-value based on 2000 replicates) on a version of this table with the row for undetermined samples removed (these are the samples that are not assigned to any cluster with p>0.8) results in a p-value <0.001.

group	*E. europaeus*	*V. opulus*	total
1	889	3	892
2	127	8	135
3	289	1	290
4	35	43	78
5	252	0	252
6	6	40	46
undet.	21	1	22
total	1619	96	1715

Table S9: Contingency table showing the number of samples per genetic group per summer host (assignments based on **STRUCTURE** under K=6). A Fisher's Exact Test (simulated p-value based on 2000 replicates) on a version of this table with the row for undetermined samples removed (these are the samples that are not assigned to any cluster with p>0.8) results in a p-value <0.001.

group	Am	Ap	As	Al	Bv	Cb	Ca	C	Ga	Gm	Mc	Pr	Ro	Tm	total
**1**	0	0	2	0	25	0	25	0	1	0	9	14	2	0	78
**2**	1	0	4	3	0	8	0	17	0	0	6	3	6	0	48
**3**	0	0	0	0	0	0	0	0	1	0	0	0	2	0	3
**4**	0	0	0	0	0	0	0	0	0	0	0	0	0	0	0
**5**	0	0	0	0	0	0	0	1	12	2	0	3	25	0	43
**6**	20	23	16	19	0	6	0	8	14	22	1	8	0	20	157
**undet.**	2	3	4	3	0	0	0	3	1	5	0	1	2	1	25
**total**	23	26	26	25	25	14	25	29	29	29	16	29	37	21	354

Legend		
	Am	*Achillea millefolium*
	Ap	*Aegopodium podagraria*
	As	*Anthriscus sylvestris*
	Al	*Arctium lappa*
	Bv	*Beta vulgaris*
	Cb	*Capsella bursa-pastoris*
	Ca	*Chenopodium album*
	C	*Cirsium spp.*
	Ga	*Galium aparine*
	Gm	*Galium mollugo*
	Mc	*Matricaria chamomilla*
	Pr	*Papaver rhoeas*
	Ro	*Rumex obtusifolius*
	Tm	*Tropaeolum majus*

Table S10: Contingency table showing the number of samples per genetic group per summer host (assignments based on **snapclust** under K=6). A Fisher's Exact Test (simulated p-value based on 2000 replicates) on a version of this table with the row for undetermined samples removed (these are the samples that are not assigned to any cluster with p>0.8) results in a p-value <0.001.

group	Am	Ap	As	Al	Bv	Cb	Ca	C	Ga	Gm	Mc	Pr	Ro	Tm	total
**1**	0	0	2	0	25	0	25	0	1	0	9	14	2	0	78
**2**	1	3	5	3	0	8	0	17	0	1	6	3	6	0	53
**3**	0	0	0	0	0	0	0	0	1	1	0	0	3	0	5
**4**	0	0	0	0	0	0	0	0	0	0	0	0	0	0	0
**5**	0	0	0	0	0	0	0	1	12	2	0	4	26	0	45
**6**	22	23	18	22	0	6	0	11	15	25	1	8	0	21	172
**undet.**	0	0	1	0	0	0	0	0	0	0	0	0	0	0	1
**total**	23	26	26	25	25	14	25	29	29	29	16	29	37	21	354

Legend
- Am *Achillea millefolium*
- Ap *Aegopodium podagraria*
- As *Anthriscus sylvestris*
- Al *Arctium lappa*
- Bv *Beta vulgaris*
- Cb *Capsella bursa-pastoris*
- Ca *Chenopodium album*
- C *Cirsium spp.*
- Ga *Galium aparine*
- Gm *Galium mollugo*
- Mc *Matricaria chamomilla*
- Pr *Papaver rhoeas*
- Ro *Rumex obtusifolius*
- Tm *Tropaeolum majus*

Table S11: Pairwise FST values (Weir & Cockerham) and 95% confidence intervals (in brackets) between sampling sites within each of the four main groups found in the winter host data with STRUCTURE under K=6 and assigning samples to a cluster if they show an assignment probability >0.8. F_{ST} values were calculated using *pairwise.WCfst*, 95% CI were estimated using *boot.ppfst* with nboot=1000 from the R package *hierfstat* (Goudet, 2005). Values whose confidence intervals do not include zero are printed in bold and red.

	Gossau	***Steinmaur***
**1-yellow**		
Faellanden	0.000 [0.000, 0.001]	0.001 [0.000, 0.002]
Gossau		0.001 [0.000, 0.002]
**2-orange**		
Faellanden	0.003 [0.000, 0.007]	0.004 [0.000, 0.007]
Gossau		**0.006 [0.003, 0.009]**
**3-violet**		
Faellanden	0.000 [-0.001, 0.000]	0.000 [-0.002, 0.001]
Gossau		-0.001 [-0.002, 0.000]
**5-blue**		
Faellanden	0.000 [-0.001, 0.001]	0.000 [-0.002, 0.002]
Gossau		0.000 [-0.002, 0.001]

Table S12: Pairwise F_{ST} values (Weir & Cockerham) and 95% confidence intervals (in brackets) between sampling time points within each of the four main groups found in the winter host data with STRUCTURE under K=6 and assigning samples to a cluster if they show an assignment probability >0.8. F_{ST} values were calculated using *pairwise.WCfst*, 95% CI were estimated using *boot.ppfst* with nboot=1000 from the R package *hierfstat* (Goudet, 2005). Values whose confidence intervals do not include zero are printed in bold and red.

	**19_springB**	**19_fall**	**20_springA**	**20_springB**	**20_fall**	**21_spring**
**1-yellow**						
19_springA	0.001 [0.000, 0.002]	**0.002 [0.001, 0.004]**	0.002 [0.001, 0.004]	0.001 [0.000, 0.002]	0.002 [0.000, 0.003]	0.000 [-0.001, 0.002]
19_springB		0.002 [0.000, 0.004]	0.002 [0.000, 0.003]	0.001 [0.000, 0.001]	0.002 [0.000, 0.004]	0.000 [-0.001, 0.001]
19_fall			0.000 [-0.001, 0.000]	0.000 [-0.001, 0.001]	0.000 [-0.001, 0.001]	0.002 [0.000, 0.003]
20_springA				0.000 [-0.001, 0.000]	0.000 [-0.001, 0.000]	0.001 [0.000, 0.002]
20_springB					0.000 [-0.001, 0.001]	0.000 [0.000, 0.001]
20_fall						0.001 [-0.001, 0.002]
**2-orange**						
19_springA	-0.003 [-0.005, 0.000]	-0.002 [-0.004, 0.001]	0.001 [-0.003, 0.006]	0.007 [-0.003, 0.019]	0.000 [-0.002, 0.003]	0.002 [-0.003, 0.009]
19_springB		-0.002 [-0.006, 0.002]	0.000 [-0.004, 0.005]	0.000 [-0.007, 0.008]	-0.001 [-0.004, 0.002]	0.007 [0.000, 0.015]
19_fall			0.000 [-0.005, 0.007]	-0.001 [-0.007, 0.007]	0.001 [-0.005, 0.006]	0.007 [0.000, 0.014]
20_springA				0.002 [-0.007, 0.012]	0.004 [-0.002, 0.011]	0.012 [-0.002, 0.032]
20_springB					0.005 [-0.001, 0.012]	**0.029 [0.015, 0.048]**
20_fall						0.002 [-0.005, 0.01]
**3-violet**						
19_springA	0.000 [-0.002, 0.003]	0.004 [-0.003, 0.013]	0.003 [0.000, 0.005]	-0.001 [-0.004, 0.001]	0.001 [-0.001, 0.004]	-0.001 [-0.002, 0.001]
19_springB		0.007 [-0.003, 0.021]	0.001 [-0.001, 0.002]	-0.001 [-0.003, 0.002]	0.001 [-0.002, 0.004]	0.000 [-0.002, 0.002]
19_fall			0.007 [0.001, 0.015]	0.001 [-0.006, 0.010]	0.002 [-0.002, 0.007]	0.004 [-0.004, 0.014]
20_springA				0.001 [-0.001, 0.003]	0.000 [-0.001, 0.002]	-0.002 [-0.003, -0.001]
20_springB					0.000 [-0.001, 0.002]	-0.003 [-0.004, -0.001]
20_fall						-0.001 [-0.003, 0.001]
**5-blue**						
19_springA	0.002 [-0.006, 0.010]	0.007 [0.000, 0.015]	0.007 [-0.002, 0.02]	0.006 [-0.001, 0.016]	**0.011 [0.002, 0.022]**	**0.008 [0.001, 0.017]**
19_springB		0.001 [-0.006, 0.009]	-0.001 [-0.006, 0.005]	-0.002 [-0.005, 0.001]	0.000 [-0.004, 0.004]	-0.001 [-0.004, 0.002]
19_fall			0.000 [-0.003, 0.004]	0.000 [-0.004, 0.003]	0.003 [-0.003, 0.012]	0.001 [-0.002, 0.005]
20_springA				0.002 [-0.002, 0.008]	0.002 [-0.001, 0.006]	0.000 [-0.001, 0.002]
20_springB					0.003 [-0.001, 0.008]	0.001 [-0.002, 0.008]
20_fall						0.001 [-0.001, 0.004]

Table S13: Results from the search for hybrid genotypes using *NewHybrids* in datasets containing 20 simulated hybrids and their parental populations (N=number of samples). Average values from 100 datasets with identical parents but newly simulated hybrids are shown. The input data consisted of those samples that were assigned to either of the parental cluster with p>0.8 in the STRUCTURE analysis under K=6 and for which data was complete for all eight markers.

parent A	parent B	hybrids total	of which simulated	detection probability
1 (yellow)	2 (orange)	18.20	18.20	91.00
1 (yellow)	3 (violet)	18.94	18.94	94.70
1 (yellow)	4 (green)	19.87	19.87	99.35
1 (yellow)	5 (blue)	19.88	19.88	99.40
1 (yellow)	6 (red)	19.42	19.42	97.10
2 (orange)	3 (violet)	10.31	10.31	51.55
2 (orange)	4 (green)	19.43	19.43	97.15
2 (orange)	5 (blue)	17.97	16.07	80.35
2 (orange)	6 (red)	17.84	17.84	89.20
3 (violet)	4 (green)	19.20	19.20	96.00
3 (violet)	5 (blue)	18.62	18.62	93.10
3 (violet)	6 (red)	17.90	17.90	89.50
4 (green)	5 (blue)	19.94	19.94	99.70
4 (green)	6 (red)	18.73	18.70	93.50
5 (blue)	6 (red)	19.63	19.63	98.15
	median	18.94	18.94	94.70
	average	18.39	18.26	91.32

Table S14: Results from the search for hybrid genotypes using *NewHybrids*. The number of "undetermined" samples for each subset corresponds to the number of samples that show $p<0.8$ for any cluster but highest probability to one of the considered parental clusters and second highest to the other.

parent A	parent B	nr. of "undetermined" samples in subset	total nr. of samples in subset	nr. of hybrids detected
1 (yellow)	2 (orange)	7	1164	**0**
1 (yellow)	3 (violet)	11	1267	**2**
1 (yellow)	4 (green)	38	1052	**37**
1 (yellow)	5 (blue)	8	1261	**3**
1 (yellow)	6 (red)	12	1188	**2**
2 (orange)	3 (violet)	17	478	**1**
2 (orange)	4 (green)	4	223	**0**
2 (orange)	5 (blue)	28	486	**20**
2 (orange)	6 (red)	5	386	**2**
3 (violet)	4 (green)	2	320	**0**
3 (violet)	5 (blue)	6	563	**2**
3 (violet)	6 (red)	1	481	**0**
4 (green)	5 (blue)	0	315	**0**
4 (green)	6 (red)	4	242	**2**
5 (blue)	6 (red)	4	481	**2**

Table S15: Aphid samples identified as putative hybrids, ordered by presumed parents, then sampling timepoint.

sample id	sampling timepoint	host plant	parents
A19_0028	Mar.19	*E. europaeus*	1-3
A20_0039	Mar.20	*E. europaeus*	1-3
A19_0008	Mar.19	*E. europaeus*	1-4
A19_0068	Mar.19	*E. europaeus*	1-4
A19_0127	Mar.19	*E. europaeus*	1-4
A19_0130	Mar.19	*E. europaeus*	1-4
A19_0131	Mar.19	*E. europaeus*	1-4
A19_0189	Mar.19	*E. europaeus*	1-4
A19_0196	Mar.19	*E. europaeus*	1-4
A19_0277	Apr.19	*E. europaeus*	1-4
A19_0296	Apr.19	*E. europaeus*	1-4
A19_0353	Apr.19	*E. europaeus*	1-4
A19_0372	Apr.19	*E. europaeus*	1-4
A19_0399	Apr.19	*E. europaeus*	1-4
A19_0401	Apr.19	*E. europaeus*	1-4
A19_0440	Apr.19	*E. europaeus*	1-4
A19_0455	Apr.19	*E. europaeus*	1-4
A20_0072	Mar.20	*E. europaeus*	1-4
A20_0095	Mar.20	*E. europaeus*	1-4
A20_0259	Apr.20	*V. opulus*	1-4
A20_0276	Apr.20	*E. europaeus*	1-4
A20_0293	Apr.20	*E. europaeus*	1-4
A20_0307	Apr.20	*E. europaeus*	1-4
A20_0343	Apr.20	*V. opulus*	1-4
A20_0349	Apr.20	*E. europaeus*	1-4
A20_0418	Apr.20	*E. europaeus*	1-4
A20_0422	Apr.20	*E. europaeus*	1-4
A20_0423	Apr.20	*E. europaeus*	1-4
A20_0441	Apr.20	*E. europaeus*	1-4
A20_0478	Apr.20	*E. europaeus*	1-4
A20_0481	Apr.20	*E. europaeus*	1-4
A20_0485	Apr.20	*E. europaeus*	1-4
A20_0486	Apr.20	*E. europaeus*	1-4
A20_0512	Apr.20	*E. europaeus*	1-4
A21_0146	Apr.21	*E. europaeus*	1-4
A21_0268	Apr.21	*E. europaeus*	1-4
A21_0272	Apr.21	*V. opulus*	1-4

sample id	sampling timepoint	host plant	parents
A21_0284	Apr.21	*V. opulus*	1-4
A21_0301	Apr.21	*V. opulus*	1-4
A19_0199	Mar.19	*E. europaeus*	1-5
A21_0103	Apr.21	*E. europaeus*	1-5
A21_0210	Apr.21	*E. europaeus*	1-5
A19_0171	Mar.19	*E. europaeus*	1-6
A20_0033	Mar.20	*E. europaeus*	1-6
A20_0112	Mar.20	*E. europaeus*	2-3
A19_0003	Mar.19	*E. europaeus*	2-5
A19_0051	Mar.19	*E. europaeus*	2-5
A19_0078	Mar.19	*E. europaeus*	2-5
A19_0211	Mar.19	*E. europaeus*	2-5
A19_0215	Mar.19	*E. europaeus*	2-5
A19_0463	Apr.19	*E. europaeus*	2-5
A19_1975	Oct.19	*E. europaeus*	2-5
A20_0036	Mar.20	*E. europaeus*	2-5
A20_0083	Mar.20	*E. europaeus*	2-5
A20_0186	Mar.20	*E. europaeus*	2-5
A20_0370	Apr.20	*E. europaeus*	2-5
A20_0405	Apr.20	*E. europaeus*	2-5
A20_0450	Apr.20	*E. europaeus*	2-5
A20_0466	Apr.20	*E. europaeus*	2-5
A20_2190	Oct.20	*E. europaeus*	2-5
A21_0129	Apr.21	*E. europaeus*	2-5
A21_0144	Apr.21	*E. europaeus*	2-5
A21_0147	Apr.21	*E. europaeus*	2-5
A21_0247	Apr.21	*E. europaeus*	2-5
A21_0261	Apr.21	*E. europaeus*	2-5
A21_0312	summer 21	*An. sylvestris*	2-6
A21_0365	summer 21	*C. vulgare*	2-6
A19_0093	Mar.19	*E. europaeus*	3-5
A19_0185	Mar.19	*E. europaeus*	3-5
A21_0248	Apr.21	*V. opulus*	4-6
A21_0287	Apr.21	*V. opulus*	4-6
A21_0501	summer 21	*T. majus*	5-6
A21_0661	summer 21	*P. rhoeas*	5-6

Table S16: Prevalence of the endosymbionts *Buchnera aphidicola* (an obligate symbiont, thus our positive control), *Hamiltonella defensa* and *Regiella insecticola* in the genetic clusters determined by STRUCTURE, and number of samples for which each symbiotypes was observed. H-R- : none of *H. defensa* or *R. insecticola*; H+ R- : only *H. defensa*, H-R+: only *R. insecticola*; H+ R+: both *H. defensa* and *R. insecticola*. Note: the number of samples per cluster (n) considered for the endosymbiont analyses is slightly lower than the total number of samples (cf. Table S4).

**cluster**	**n**	symbiont prevalence			# samples per symbiotype			
		Buchnera	***Hamiltonella***	***Regiella***	**H- R-**	**H+ R-**	**H- R+**	**H+ R+**
1	954	1	0.336	0.078	563	317	70	4
2	166	1	0.012	0.03	159	2	5	0
3	279	1	0.140	0.918	9	14	231	25
4	38	1	1.000	0.026	0	37	0	1
5	273	1	0.004	0.015	268	1	4	0
6	194	1	0.046	0.021	181	9	4	0
1×4 h.	37	1	0.405	0.054	20	15	2	0
2×5 h.	20	1	0.000	0.050	19	0	1	0

Table S17: Values and from pairwise Fisher's Exact tests to assess the statistical significance of differences in symbiotypes (see Table S15) between the genetic groups inferred from the STRUCTURE K=6 solution. The Bonferroni-corrected significance level is 0.05/18 = 0.00278. Six

comparison	p-value Fisher's Exact Test
1-yellow vs 2-orange	<0.000001
1-yellow vs 3-violet	<0.000001
1-yellow vs 4-green	<0.000001
1-yellow vs 5-blue	<0.000001
1-yellow vs 6-red	<0.000001
2-orange vs 3-violet	<0.000001
2-orange vs 4-green	<0.000001
2-orange vs 5-blue	0.281171
2-orange vs 3-red	0.140102
3-violet vs 4-green	<0.000001
3-violet vs 5-blue	<0.000001
3-violet vs 4-red	<0.000001
4-green vs 5-blue	<0.000001
4-green vs 6-red	<0.000001
5-blue vs 6-red	0.004436
1-yellow vs 1-4 hybrids	0.779100
4-green vs 1-4 hybrids	<0.000001
2-orange vs 2-5 hybrids	0.604900
5-blue vs 2-5 hybrids	0.350200

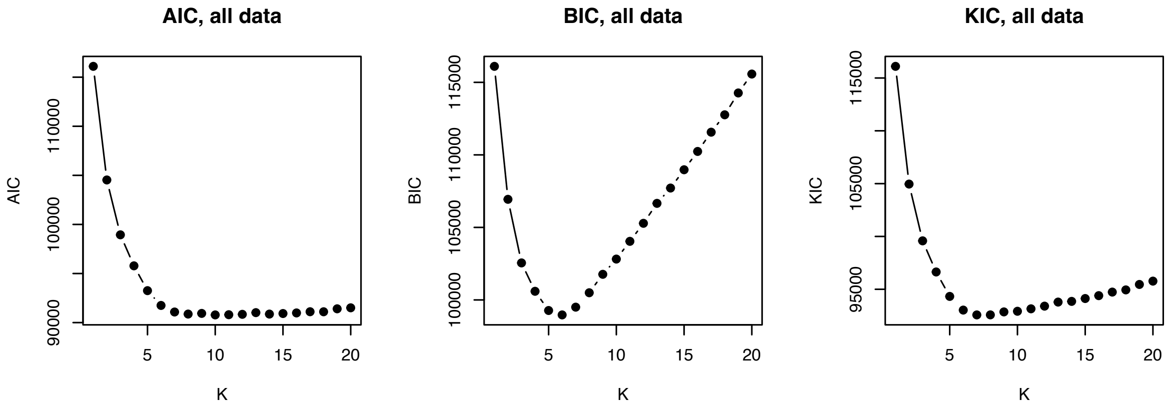

Figure S1: AIC, BIC and KIC values for the clustering results obtained with *snapclust* applied to the full dataset (2099 samples) for different numbers of clusters (K). Minimal values or "elbows" in the curves, i.e. trends that change from decreasing to increasing, may hint at the "optimal" number of clusters in the data, a such is most clearly visible in the BIC plot at K=6.

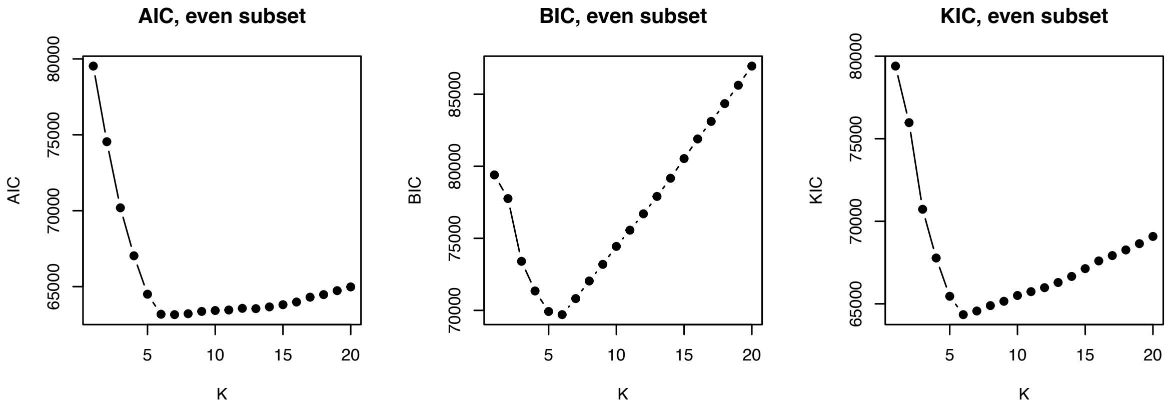

Figure S2: AIC, BIC and KIC values for the clustering results obtained with *snapclust* applied to the more balanced data subsets (containing a subset of data from the largest cluster such as to have more similar numbers of samples belonging to the six clusters initially inferred with *snapclust*), for different numbers of clusters (K).

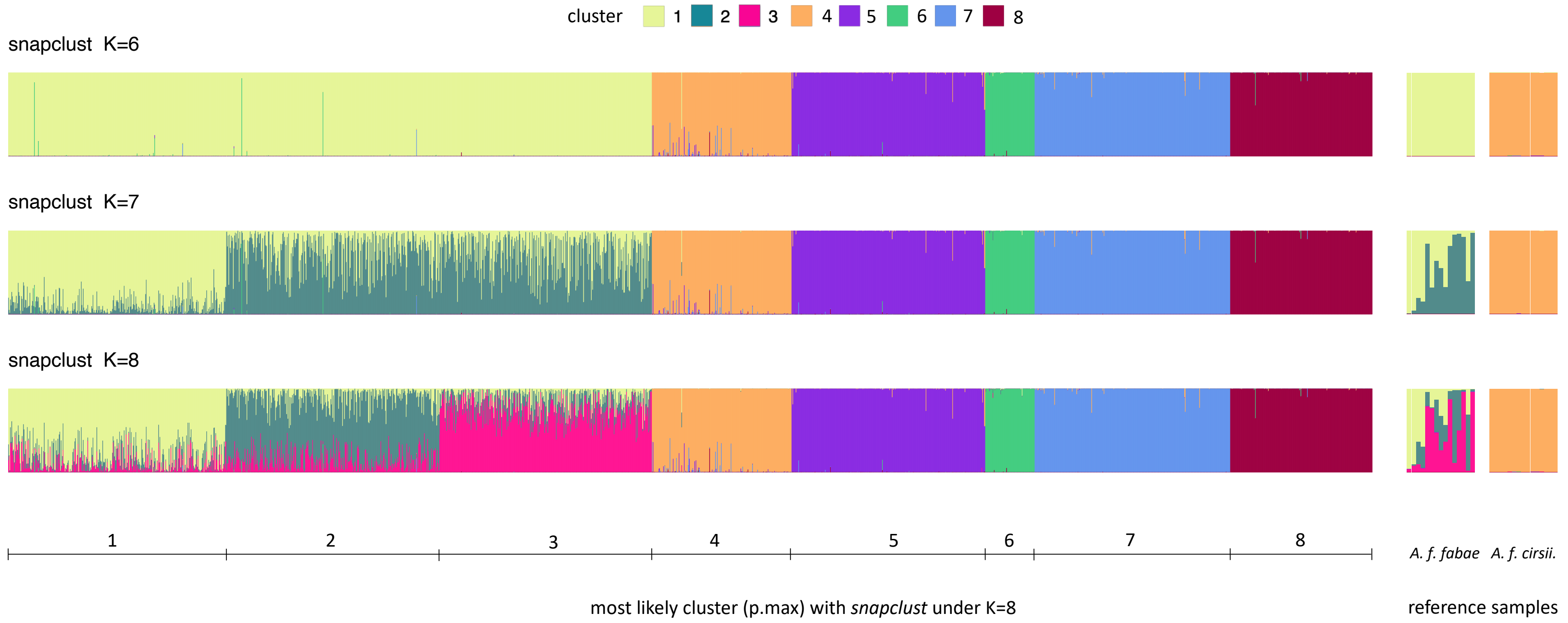

Figure S3: Clustering results from *snapclust* for the number of clusters K=6, K=7, or K=8. Each aphid individual is represented by a vertical bar, the proportion of this bar in a specific color represents the likelihood that the sample belongs to the respective cluster (membership probability). For each K, the wide boxes to the left show all 2099 samples used in the analysis. For all solutions the samples are ordered according to the cluster for which they show highest membership probability in the K=8 result. The two narrow boxes to the right zoom in on the reference samples known to represent *A. f. fabae* and *A. f. cirsiiacanthoides*, respectively.

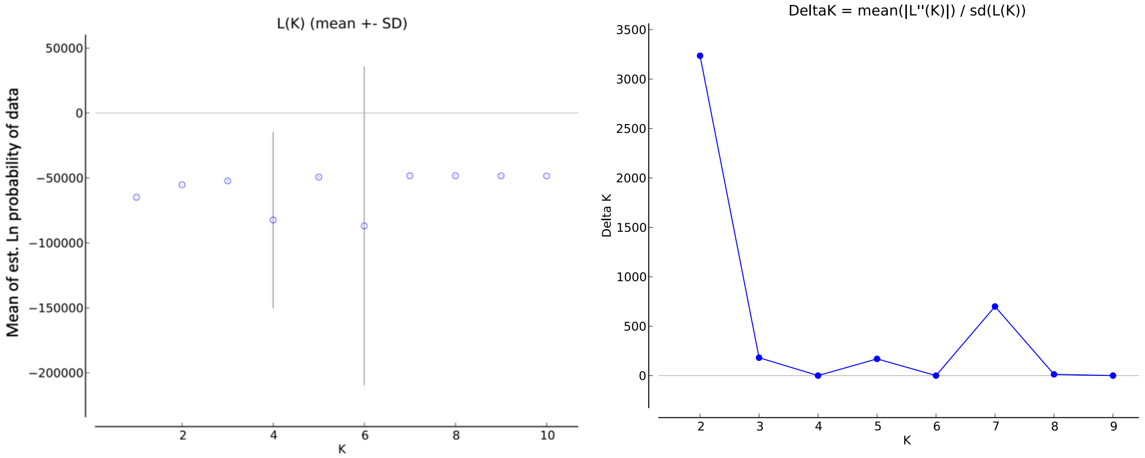

K	Reps	Mean LnP(K)	Stdev LnP(K)	Ln'(K)	lLn''(K)l	Delta K
1	10	-64852.410000	0.202485	—	—	—
2	10	-55207.730000	2.063465	9644.680000	6677.510000	3236.066108
3	10	-52240.560000	182.159059	2967.170000	33101.980000	181.720197
4	10	-82375.370000	67543.718046	-30134.810000	63197.520000	0.935654
5	10	-49312.660000	415.680816	33062.710000	70636.900000	169.930623
6	10	-86886.850000	122394.920388	-37574.190000	76213.340000	0.622684
7	10	-48247.700000	55.195733	38639.150000	38625.440000	699.790332
8	10	-48233.990000	7.946131	13.710000	107.830000	13.570126
9	10	-48328.110000	5.843600	-94.120000	4.670000	0.799165
10	10	-48426.900000	15.261426	-98.790000	—	—

Figure S4: Output from STRUCTURE HARVESTER (Evanno *et al.*, 2005) used to determine the optimal number of clusters (K) in the results from running STRUCTURE on the **full dataset** using the settings suggested by Wang (2017). These settings should improve the detection of small clusters in datasets with uneven or unknown samples sizes, but they may also lead to overestimation of the "optimal" number of clusters. The "optimal" number of clusters might be derived from the plot on the left as the K (y-axis) for which the mean Ln of assignment probability (x-axis) is highest, or sometimes also where the curve flattens down (Pritchard *et al.*, 2000), no such pattern can be seen here. From the plot on the right, the optimal number of K (y-axis) might be derived as the one where DeltaK is maximal (x-axis, Evanno method, Evanno *et al.*, 2005), i.e. K = 2 is determined as the optimal number of clusters here. The summary table shows the values that are visualized in the plot.

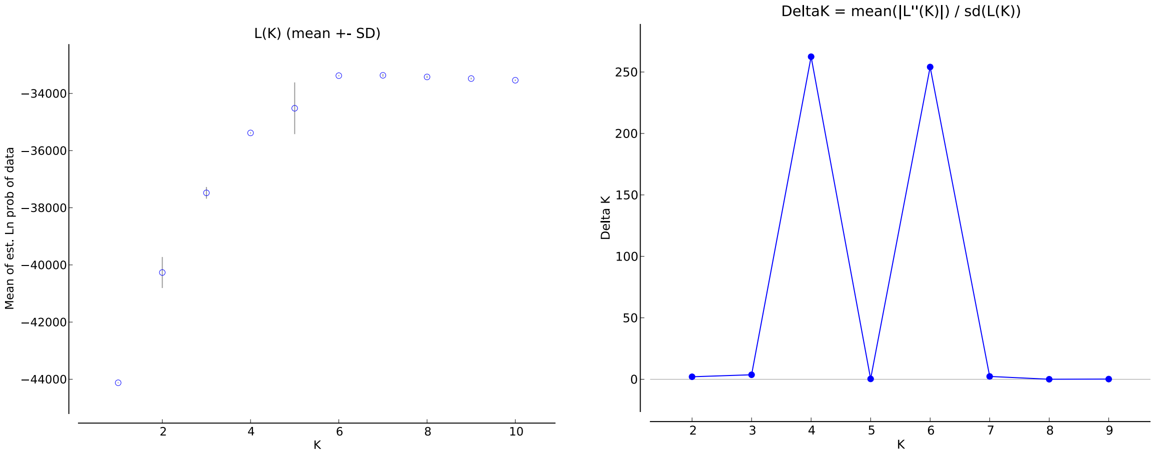

K	Reps	Mean LnP(K)	Stdev LnP(K)	Ln'(K)	|Ln''(K)|	Delta K
1	10	-44123.250000	0.143372	—	—	—
2	10	-40265.540000	526.151973	3857.710000	1070.600000	2.034773
3	10	-37478.430000	188.260570	2787.110000	689.730000	3.663699
4	10	-35381.050000	4.704194	2097.380000	1234.640000	262.455143
5	10	-34518.310000	888.232338	862.740000	277.870000	0.312835
6	10	-33377.700000	4.442722	1140.610000	1128.810000	254.080725
7	10	-33365.900000	29.399131	11.800000	68.070000	2.315375
8	10	-33422.170000	24.820916	-56.270000	0.710000	0.028605
9	10	-33479.150000	12.176685	-56.980000	2.020000	0.165891
10	10	-33538.150000	8.287910	-59.000000	—	—

Figure S5: Output from STRUCTURE HARVESTER (Evanno *et al.*, 2005) used to determine the optimal number of clusters (K) in the results from running STRUCTURE on the more **balanced data subset** with the settings suggested by Wang (2017). These settings should improve the detection of small clusters in datasets with uneven or unknown samples sizes, but they may also lead to overestimation of the "optimal" number of clusters. The "optimal" number of clusters might be derived from the plot on the left as the K (y-axis) for which the mean Ln of assignment probability (x-axis) is highest, or sometimes also where the curve flattens down (Pritchard *et al.*, 2000), which is the case at K=6 here. From the plot on the right, the optimal number of K (y-axis) might be derived as the one where DeltaK is maximal (x-axis, Evanno method, Evanno *et al.*, 2005). The two peaks at K = 4 or K=6 visible in this plot are caused by the high uncertainty for the K=5 solution visible in the plot on the left.

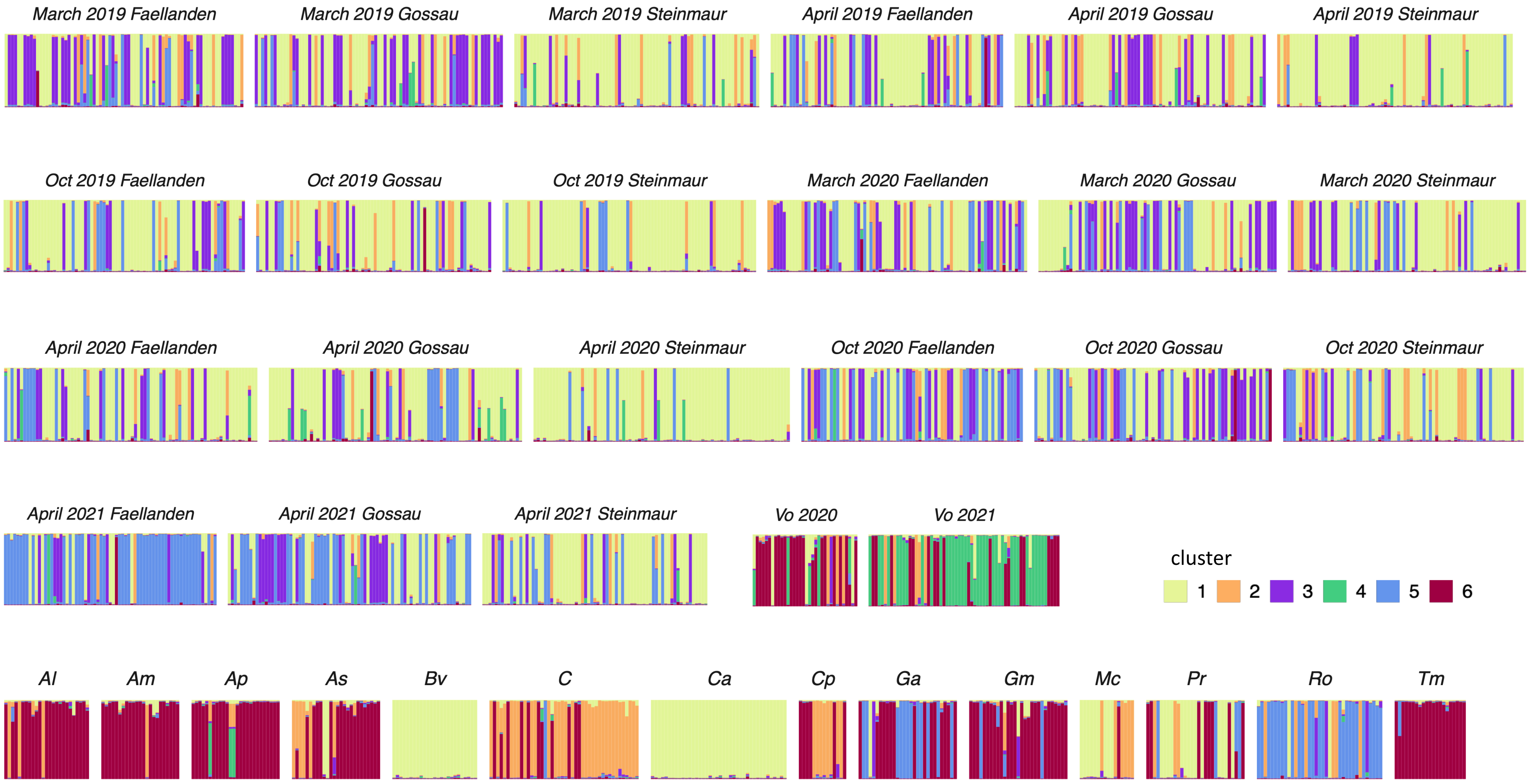

Figure S6: Clustering results from STRUCTURE under K=6. Each aphid individual is represented by a vertical bar, the proportion of this bar in a specific color represents the likelihood that the sample belongs to the respective cluster (membership probability). The plot shows the same results (bars) as the second plot in Figure 1, but ordered by sampling timepoint here (first four rows, host plant = *Euonymus europaeus* if not indicated differently) and/or host plant (last row). Host plant abbreviations: Am *Achillea millefolium*, Ap *Aegopodium podagraria*, As *Anthriscus sylvestris*, Al *Arctium lappa*, Bv *Beta vulgaris*, Cb *Capsella bursa-pastoris*, Ca *Chenopodium album*, C *Cirsium spp*., Ga *Galium aparine*, Gm *Galium mollugo*, Mc *Matricaria chamomilla*, Pr *Papaver rhoeas*, Ro *Rumex obtusifolius*, Tm *Tropaeolum majus,* Vo *Viburnum opulus.*

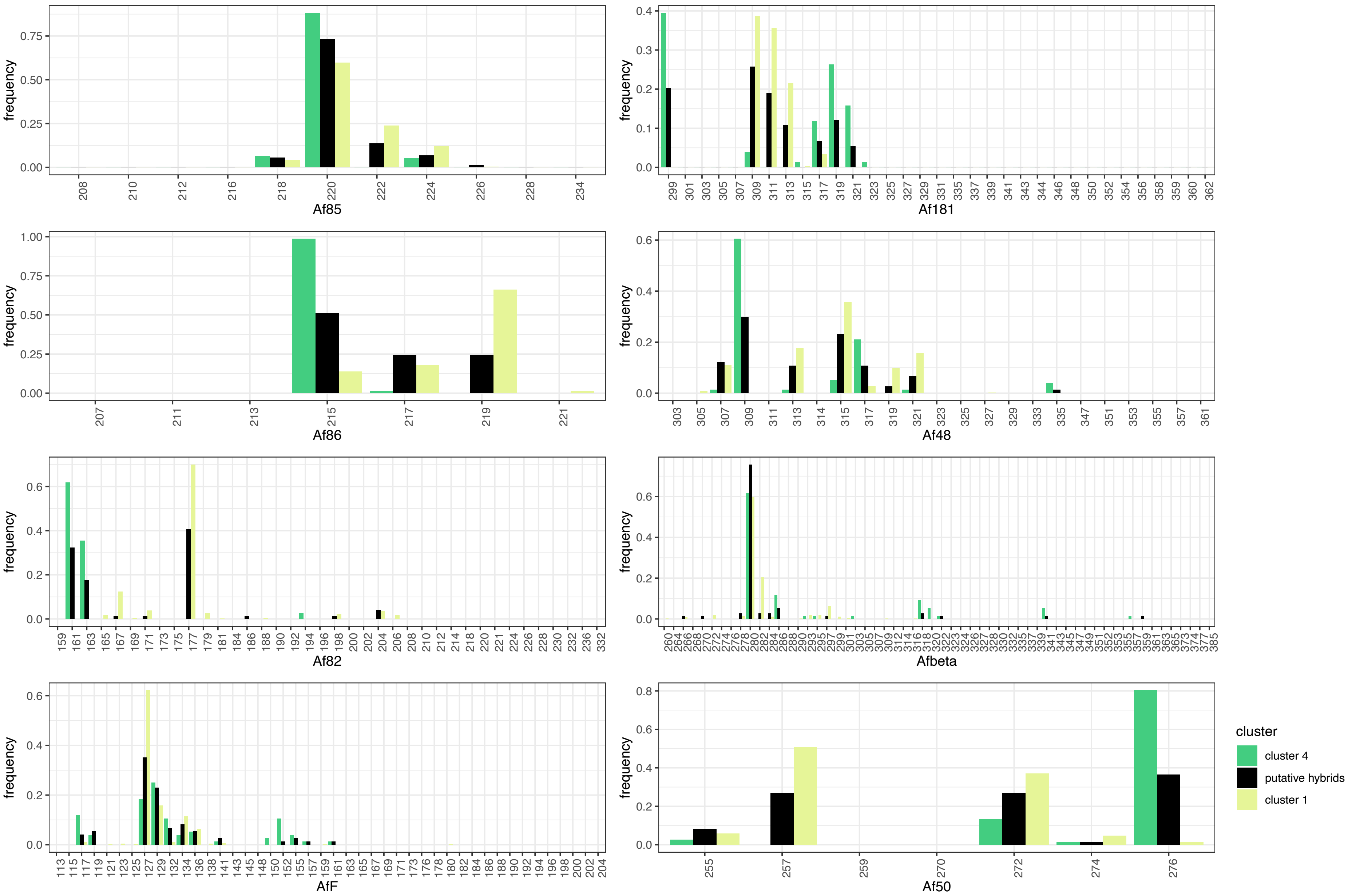

Figure S7: Allele frequencies in cluster 1 (yellow, *A. f. fabae*) and cluster 4 (green, putative *A. viburni*), and their putative hybrids (black).

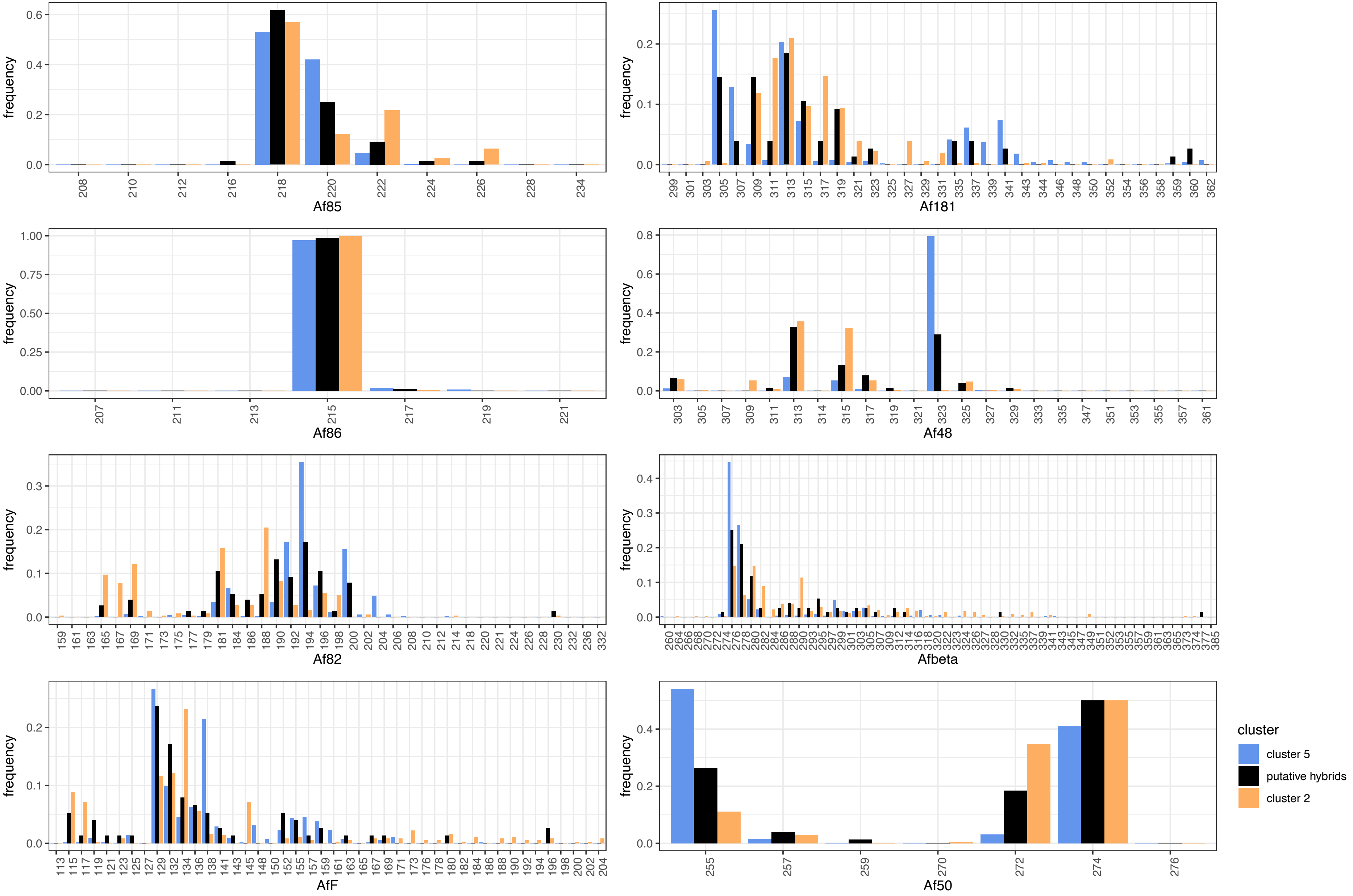

Figure S8: Allele frequencies in cluster 2 (orange, *A. f. cirsiiacanthoidis*) and cluster 5 (blue), and their putative hybrids (black).

Supplementary analysis: genetic clustering using DAPC

We compared the results of three different clustering methods to assess the genetic structure present in our data and to assign samples to genetic clusters: *snapclust* (Beugin *et al.*, 2018), STRUCTURE (Falush *et al.*, 2003; Pritchard *et al.*, 2000) and DAPC (Jombart *et al.*, 2010). As the results were very similar, we only present the *snapclust* and STRUCTURE analyses in the main manuscript. Methods and results of the DAPC analysis are summarized here.

Methods

For DAPC, initial groups used as input need to be defined in a preceding step. To do so, we used the k-means algorithm implemented in the *adegenet* function *find.clusters*, retaining all PCs. The function provides BIC values that can be used to assess goodness-of-fit for different clustering solutions (Jombart *et al.*, 2010). For the subsequent DAPC analyses we generally retained all eigenvalues as recommended by the authors for small numbers of clusters (Jombart *et al.*, 2010), and we used cross validation with *adegenet::xvalDapc* and default settings to decide on the number of principal components to retain. When applying k-means to the full dataset to obtain input clusters for DAPC, BIC values hint at K=2 as the optimal number of clusters (Figure S10A). Carrying out DAPC for K=2 (10 PCs retained), the resulting split corresponds to a separation of the largest cluster inferred by *snapclust* and STRUCTURE from all other samples. Within this largest cluster, BIC values resulting from k-means clustering suggest no further substructure (Figure S10B). As K=2 is clearly an insufficient subdivision, and since also DAPC showed to be sensitive to unequal sample sizes, we focused the further DAPC analysis on the more balanced dataset as described in the main manuscript.

Results

Using DAPC to cluster the more balanced dataset, BIC values indicate K=6 as the optimal number of clusters (Figure S10C). Under K=6, the group assignments resulting from DAPC (20 PCs retained) are very similar to the ones obtained with *snapclust* or STRUCTURE (Figure S11). Under K=7 (20 PCs retained), a different cluster than with either *snapclust* or STRUCTURE gets subdivided further (cluster 5, blue), albeit with low confidence of assignment, which additionally argues for K=6 as the optimal solution. Assuming six clusters, all but clusters 2 (orange) and 3 (violet) get clearly separated along the first two linear discriminants (Figure S10). Clusters 2 and 3 are separated along the 3rd and 5th linear discriminants, while the 4th discriminant is mainly separating cluster 4 (green) from all other samples (Figure S11).

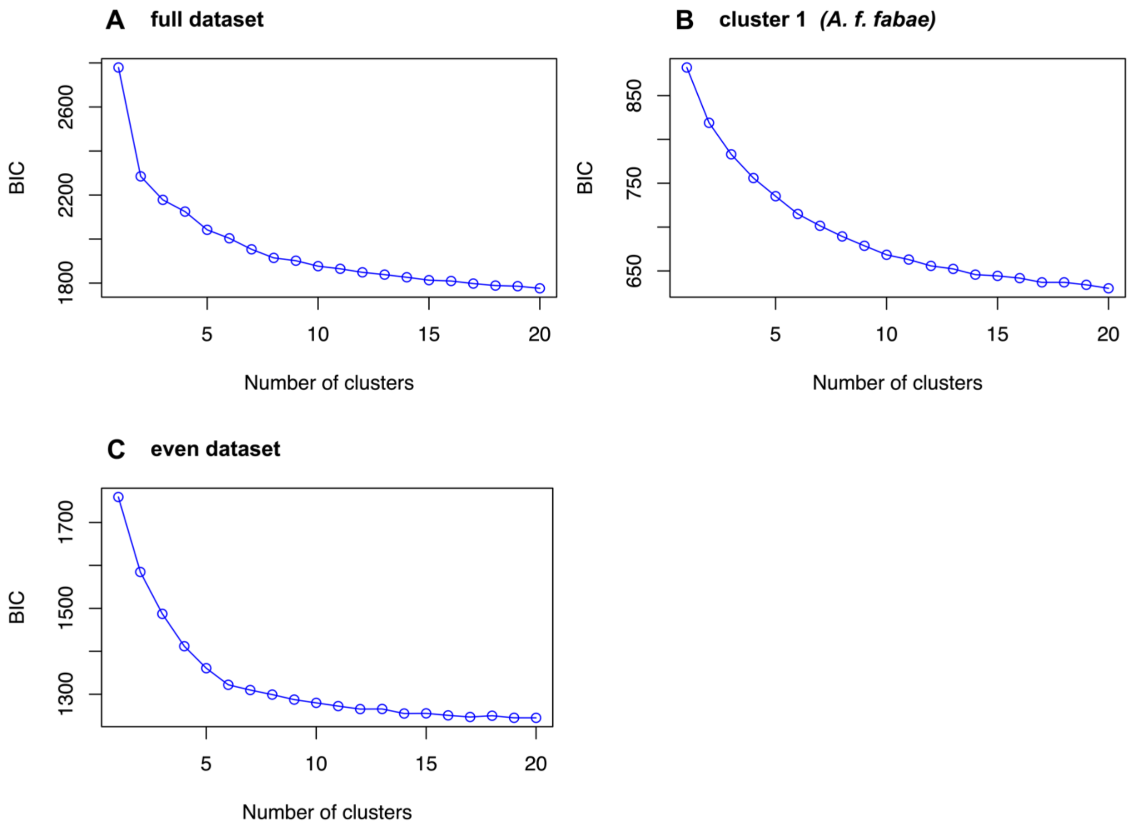

Figure S9: BIC values for k-means clustering solutions on (a) the full dataset, K=2 is suggested here; (b) on samples belonging to the presumed *A. f. fabae* cluster (cluster 1) only, no substructure is suggested here; (c) on the even data subset, K=6 is suggested here (the "optimal" number of clusters might be indicated by minimal values and/or an "elbow" in the curve).

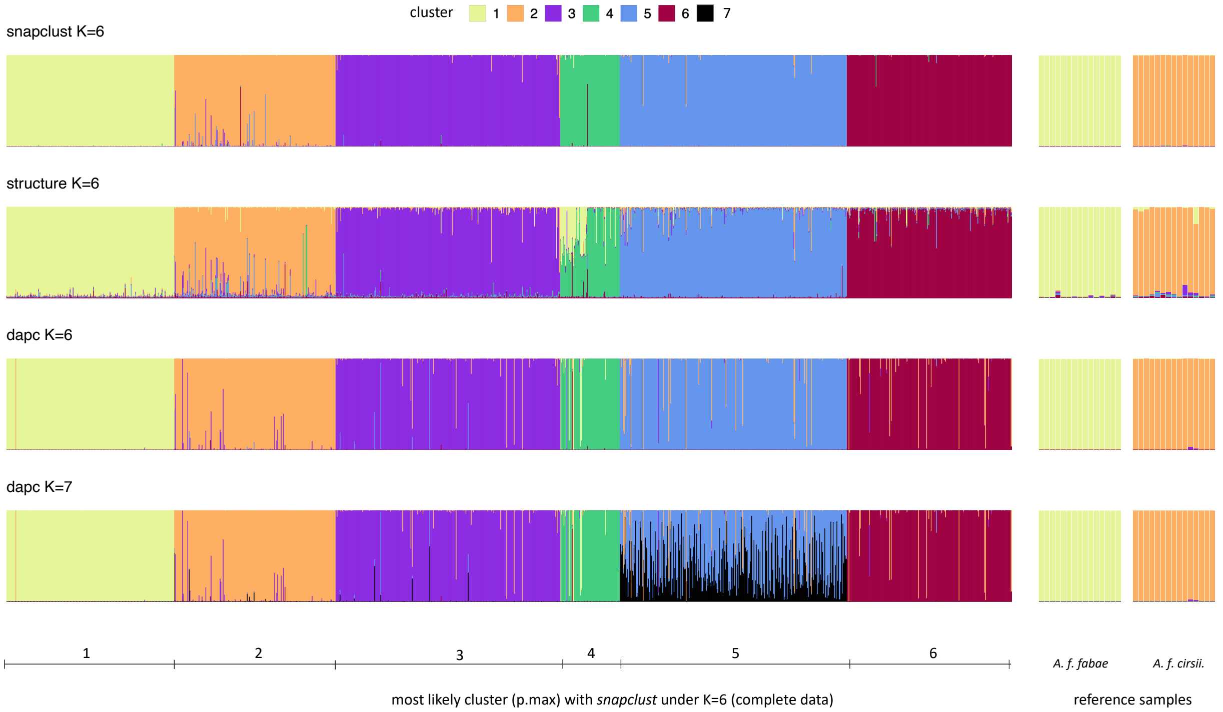

Figure S10: Clustering results from *snapclust* (sc), STRUCTURE (STR) and DAPC applied to the more **balanced data subset**. Each aphid individual is represented by a vertical bar, the proportion of this bar colored in a specific color corresponds to the likelihood that the sample belongs to a specific cluster (membership probability). For each K, the wide boxes to the left show **all 1333 samples** used in the analysis next to each other. For all solutions the samples are ordered by their most likely cluster in the *snapclust* K=6 solution in the full data analysis. The two narrow boxes to the right zoom in on the reference samples from *A. f. fabae* and *A. f. cirsiiacanthoides*, respectively.

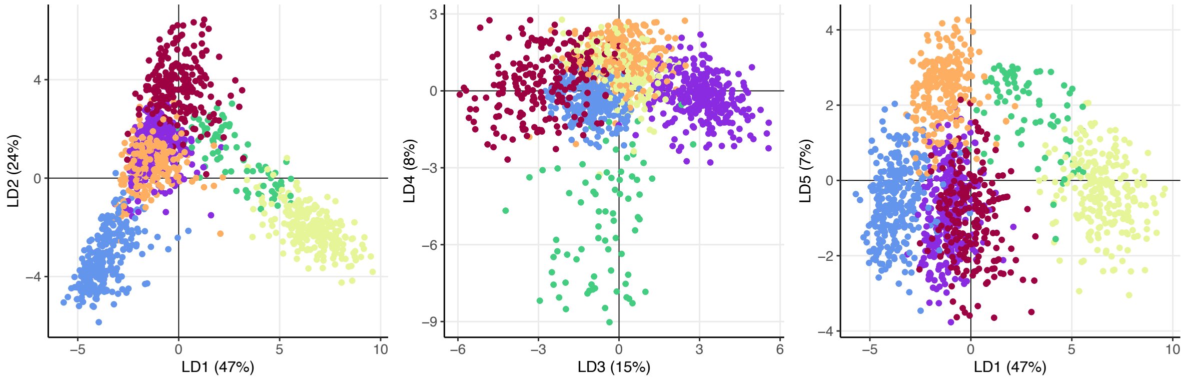

Figure S11: Discriminant analysis of principal components (DAPC) after dividing the more balanced data subset into six groups using the k-means algorithm. The axes represent the 1st and 2nd (left), the 3rd and 4th (middle) or the 1st and 5th (right) linear discriminants; the number in brackets shows the percentage of variance explained by the discriminant. Each dot represents an individual aphid, its color corresponds to its group assignment as used for DAPC (which is very similar but not identical to the group assignment resulting by the *snapclust* and STRUCTURE analyses).

References

Beugin, M. P., Gayet, T., Pontier, D., Devillard, S., & Jombart, T. (2018). A fast likelihood solution to the genetic clustering problem. *Methods Ecol. Evol., 9*(4), 1006-1016. https://doi.org/10.1111/2041-210X.12968

Blackman, R. L., & Eastop, V. F. (2000). *Aphids on the world's crops: an identification and information guide*. Chichester: John Wiley & Sons Ltd.

Blackman, R. L., & Eastop, V. F. (2017). Taxonomic issues. In *Aphids as crop pests* (pp. 1-36): CABI Wallingford UK.

Coeur d'acier, A., Sembène, M., Audiot, P., & Rasplus, J. Y. (2004). Polymorphic microsatellites loci in the black aphid, *Aphis fabae* Scopoli, 1763 (Hemiptera, Aphididae). *Mol. Ecol. Notes, 4*(2), 306-308. https://doi.org/10.1111/j.1471-8286.2004.00652.x

Evanno, G., Regnaut, S., & Goudet, J. (2005). Detecting the number of clusters of individuals using the software STRUCTURE: a simulation study. *Mol. Ecol., 14*(8), 2611-2620. https://doi.org/10.1111/j.1365-294X.2005.02553.x

Falush, D., Stephens, M., & Pritchard, J. K. (2003). Inference of population structure using multilocus genotype data: linked loci and correlated allele frequencies. *Genetics, 164*(4), 1567-1587. https://doi.org/10.1093/genetics/164.4.1567

Ferrari, J., West, J. A., Via, S., & Godfray, H. C. (2012). Population genetic structure and secondary symbionts in host-associated populations of the pea aphid complex. *Evolution, 66*(2), 375-390. https://doi.org/10.1111/j.1558-5646.2011.01436.x

Gimmi, E., Wallisch, J., & Vorburger, C. (2023). Defensive symbiosis in the wild: Seasonal dynamics of parasitism risk and symbiont-conferred resistance. *Mol. Ecol., 32*(14), 4063– 4077. https://doi.org/10.1111/mec.16976

Goudet, J. (2005). Hierfstat, a package for R to compute and test hierarchical F-statistics. *Mol. Ecol. Notes, 5*(1), 184-186. https://doi.org/10.1111/j.1471-8286.2004.00828.x

Hafer-Hahmann, N., & Vorburger, C. (2020). Parasitoids as drivers of symbiont diversity in an insect host. *Ecol. Lett., 23*(8), 1232-1241. https://doi.org/10.1111/ele.13526

Jombart, T., Devillard, S., & Balloux, F. (2010). Discriminant analysis of principal components: a new method for the analysis of genetically structured populations. *BMC Genetics, 11*(1), 94. https://doi.org/10.1186/1471-2156-11-94

Pritchard, J. K., Stephens, M., & Donnelly, P. (2000). Inference of population structure using multilocus genotype data. *Genetics, 155*(2), 945-959. https://doi.org/10.1093/genetics/155.2.945

Wang, J. (2017). The computer program structure for assigning individuals to populations: easy to use but easier to misuse. *Mol. Ecol. Resour., 17*(5), 981-990. https://doi.org/10.1111/1755-0998.12650
